# Polygonatum odoratum extract ameliorates acetaminophen-induced acute liver injury by modulating the gut-liver axis and remodeling microbial and ceramide metabolism to suppress hepatic ferroptosis: insights from multi-omics

**DOI:** 10.3389/fphar.2026.1907173

**Published:** 2026-07-29

**Authors:** Huanghui Qin, Runxiao Chen, Ying Shi, Caibin Li, Yiming Bin, Zhaoming Zeng, Bin Deng, Yubo Xiao, Lanyu Li

**Affiliations:** 1 Guangxi Key Laboratory of Diabetic Systems Medicine, Guilin Medical University, Guilin, Guangxi, China; 2 Engineering Technology Research Center of Natural Medicine R&D for Prevention and Treatment of Metabolic Chronic Diseases in Huaihua City, Huaihua, Hunan, China; 3 Guangxi Key Laboratory of Molecular Medicine in Liver Injury and Repair, The First Affiliated Hospital of Guilin Medical University, Guilin, Guangxi, China; 4 Hunan Provincial Key Laboratory for Synthetic Biology of Traditional Chinese Medicine, School of Medical Laboratory Science,Hunan University of Medicine, Huaihua, Hunan, China

**Keywords:** ceramide, ferroptosis, gut microbiota, gut-liver axis, liver injury, *Polygonatum odoratum* extract

## Abstract

**Context:**

Drug-induced liver injury remains a major clinical challenge due to the lack of effective therapeutic options. *Polygonatum odoratum* extract (PO), derived from the traditional medicinal and edible plant *Polygonatum odoratum (Mill.) Druce*, has long been used in folk practices in forms such as herbal soups and tea infusions. However, its protective effect against acetaminophen (APAP)-induced liver injury has not yet been investigated.

**Objective:**

This study evaluated the preventive effects of PO against APAP-induced acute liver injury in mice.

**Materials and Methods:**

An APAP-induced acute liver injury model was established in C57BL/6 mice. The hepatoprotective effects of PO were validated via histopathological analysis, oxidative stress assays,and serum biomarkers Multi-omics approaches, including 16 S rDNA sequencing, transcriptomics, and network pharmacology, were integrated with molecular biology techniques and pseudo-germ-free (PGF) mouse models to elucidate the underlying mechanisms.

**Results:**

PO significantly alleviated hepatic histopathological damage, oxidative stress, and reduced serum ALT, AST, and LDH. PO remodeling gut microbiota, mitigated colonic inflammation and oxidative stress, and improved intestinal morphology, effects abolished by microbiota depletion. Mechanistically, PO maybe inhibited hepatic ferroptosis via the gut microbiota–ceramide axis, evidenced by upregulation of GPX4 and FSP1, NRF2/KEAP1 pathway activation, downregulation of ACSL4, TFRC/TFR2, and DUOX2, restored iron homeostasis, and decreased lipid peroxidation and ROS. *In vitro*, PO demonstrated anti-ferroptotic effects in APAP- or RSL3-treated AML12 cells.

**Discussion and Conclusion:**

PO confers hepatoprotection via the gut–liver axis, which is associated with changes in ceramide metabolism and ferroptosis-related markers.

## 1 Introduction

Drug-induced liver injury (Mao, 2023) represents one of the most prevalent and challenging clinical complications in pharmaceutical development. Furthermore, several approved therapeutics have been withdrawn from the market due to idiosyncratic or intrinsic hepatotoxicity (Li et al., 2022). Acetaminophen (APAP), a widely used analgesic and antipyretic agent, is a leading cause of acute DILI, particularly in cases of overdose (Hosack et al., 2023). According to the 2017 FDA report, APAP overdose accounts for 46% of acute liver failure cases in the United States, with ≥500 annual fatalities attributed to its hepatotoxicity (Ghanem et al., 2016; Ramachandran et al., 2024).

The pathogenesis of APAP-induced liver injury reportedly involves cytochrome P450 (CYP)-mediated metabolic activation, primarily via CYP2E1, which converts APAP into the highly reactive metabolite N-acetyl-p-benzoquinone imine (NAPQI). Under physiological conditions, NAPQI is detoxified by GSH. However, APAP overdose depletes hepatic GSH reserves, leading to NAPQI accumulation and covalent binding to cellular proteins. This triggers oxidative stress, mitochondrial dysfunction, lipid peroxidation, and ferroptosis, a regulated cell death pathway driven by iron-dependent accumulation of lipid ROS, ultimately culminating in hepatocyte necrosis (Akakpo et al., 2021; Liao et al., 2023). Given the narrow therapeutic window of N-acetylcysteine (NAC), the current clinical gold standard, developing novel therapeutic strategies to mitigate APAP-induced liver injury remains a critical unmet need (Coskun et al., 2021; Shen et al., 2019; Sun et al., 2022).

*Polygonatum odoratum* Extract (PO) is derived from the traditional medicinal and edible plant Polygonatum odoratum (known as “Yuzhu” in Chinese) (Zhou et al., 2015). Its key bioactive components include polygonatum polysaccharides, steroidal saponins, and flavonoids, which contribute to various physiological effects such as antioxidant activity, immune regulation, and moisturizing properties (Wang et al., 2009; Zhou et al., 2015). *Polygonatum odoratum (Mill.) Druce*, the dried rhizome of the Liliaceae family, is listed among China’s first batch of dual-purpose medicinal and edible herbs in the food and medicine homology catalog (Cong, 2024; Liu et al., 2025). Historically, the Yellow Emperor’s Inner Canon of Medicine documented PO as “sweet in property, capable of tonifying, harmonizing, and moderating”, highlighting its traditional use to nourish yin, moisten dryness, and promote fluid production. In folk practices, it has long been used in dietary therapy through forms like herbal soups and tea-based dishes, embodying the culinary wisdom of “moisturizing without greasiness, nourishing without causing dryness.“Modern food industry employs standardized extraction technologies to convert it into a stable raw material, now widely utilized in functional beverages, baked goods, and natural preservatives (Sun-Waterhouse et al., 2024).

Despite the widespread application of PO in multi-herb formulations and dietary therapies for conditions such as hyperlipidemia and hyperglycemia, its potential in liver diseases remains underexplored. We sought to explore its preventive potential in acute liver injury.

Sphingolipids, as bioactive secondary messengers, play a pivotal role in regulating critical intercellular communication pathways, and their metabolic modulation is closely associated with inflammatory processes and oxidative stress responses (Spaulding and Bollag, 2022; Zhang et al., 2022) While current research on sphingolipids has primarily focused on metabolic diseases, the liver, the central metabolic organ in humans, has recently emerged as a research hotspot for investigating the relationship between sphingolipid dysregulation and the pathogenesis and progression of liver diseases (Di Sessa et al., 2021; Jackson et al., 2022). Our preliminary studies have demonstrated the significant involvement of sphingosine-1-phosphate in APAP-induced liver injury; however, the broader role of sphingolipid signaling pathways in hepatic damage remains relatively underexplored (Qin et al., 2025). Among sphingolipids, ceramide stands out as the most bioactive metabolite, contributing to the formation of membrane microdomains known as “lipid rafts”. Accumulating evidence underscores its close association with the onset and prognosis of various diseases, particularly those linked to oxidative stress, lipid peroxidation, and inflammatory cascades (Custodia et al., 2021; Devulapalli, 2025; Ueda, 2022).

Ferroptosis, a novel form of regulated cell death first identified in 2012, is now understood to be distinct from conventional apoptosis, necrosis, and autophagy. It is characterized by lethal lipid peroxidation driven by an imbalance between the generation and degradation of intracellular lipid reactive oxygen species (ROS), accompanied by iron accumulation (Dixon and Olzmann, 2024; Rochette et al., 2022). Morphologically, ferroptosis manifests as mitochondrial shrinkage and a reduction (or even loss) of mitochondrial cristae, processes closely linked to oxidative stress and inflammation (Xiang et al., 2025).

Current evidence highlights that three key pathways mediate ferroptosis: iron metabolism, redox homeostasis, and lipid metabolism. In the context of acute liver injury, dysregulation of the redox system, iron overload, and/or peroxidation of polyunsaturated fatty acids (PUFAs) can trigger ferroptosis. Consequently, pharmacological inhibition of ferroptosis has emerged as a promising therapeutic strategy for APAP-induced liver injury (Bi et al., 2024; Liang et al., 2022; Rochette et al., 2022; Yan et al., 2021).

In this study, we sought to further elucidate the interplay among PO, the gut microbiota, ceramide metabolism, and ferroptosis, thereby uncovering the mechanistic basis and molecular targets by which PO confers protection against APAP-induced acute liver injury in mice.

## 2 Materials and methods

### 2.1 Material

*Polygonatum odoratum* extract (derived from dried rhizomes of *Polygonatum odoratum*, supplied by Hunan Mingshun Pharmaceutical Co.) The extraction process was as follows: 1) Raw material identification was performed via morphological identification and HPLC fingerprint comparison. 2) After screening through a 40-mesh sieve, the material was ground at low temperature (≤40 °C), compliant with Chinese Pharmacopoeia standards (moisture ≤12%, ash ≤5%). 3) Dynamic countercurrent extraction was performed using water-ethanol (70:30, v/v) at 80 °C with a solid-to-liquid ratio of 1:15, extracted three times. The filtrate was concentrated under reduced pressure to a density of 1.10–1.15.4) Purification was carried out using D101 macroporous resin (30% ethanol elution), followed by spray drying (air inlet temperature 180 °C, air outlet temperature 85 °C) to obtain powder. 5) Standardized indicators included: polysaccharides ≥30% (phenol-sulfuric acid method, as glucose), flavonoids ≥5% (UV spectrophotometry, as rutin), and saponins ≥1.2% (HPLC-ELSD, Polygonatoside). Quality control was maintained by near-infrared spectroscopy (NIRS) to monitor batch consistency. Acetaminophen (MCE); Mouse IL-1beta (E-EL-M0037,Elabscience) Mouse IL-6 double antibody sandwich ELISA detection kit (KE10007,Proteintech); Mouse TNF-α double antibody sandwich ELISA detection kit (KE10002, Proteintech); Aspartate transaminase test kit (C010-2–1,Jiancheng Biotechnology, Nanjing); Alanine aminotransferase assay kit (Mindray, Shenzhen); Lactate dehydrogenase assay kit (Mindray, Shenzhen); Trace Reduced Glutathione Assay Kit (A006-2–1,Jiancheng Nanjing); Malondialdehyde test kit (A003-1,Jiancheng Nanjing); Superoxide dismutase assay kit (A001-3,Jiancheng Nanjing); 4-HNE ELISA KIT (JRXW2004266 Ruixin Biotech); Ferrous Iron Colorimetric Assay Kit (E-BC-K773-M,elabscience); Mouse *FTH* ELISA KIT (EM30738XS,WEIAOBI); FE (RXW202595M, Ruixin Biotech); TFR (XY9M2280,X-Y Biotechnology); Mouse TFR2 ELISA KIT (RXW202242 M,Ruixin Biotech); Protein marker (26,616,Thermo Fisher)NRF2 Antibody (T55136,Abmart); Anti-ACSL4 (A04372-2,BOSTER); Anti-FPN1 (A01953-2, BOSTER); KEAP1 (80744-1-RR,Proteintech); p-P65 (3033T, cell signaling); P65 (8242T, cell signaling); P62 (T55546S, Abmart); SLC7A11 (A03036-2,BOSTER); NQO1 Ab (CY6710,abways); HO-1 (CY8761, abways, Shanghai); Anti-GPX4 (BM5231 BOSTER); LC3B (T55992 S,Abmart); Reactive oxygen species fluorescence test kit (E-BC-K138-F,Elabscience); FSP1(M06541,BOSTER); DUOX2 (A02186,BOSTER); LacCer(IVDUCT); PERK (3192 T,cell signaling); AMPK(TR19913,Abmart).

### 2.2 PO treatment and construction of APAP-induced acute liver injury model in mice

All animal procedures were conducted in accordance with the Guide for the Care and Use of Laboratory Animals issued by the National Institutes of Health (USA) and the National Standard for Laboratory Animal Welfare Ethical Review of the People’s Republic of China. Experimental protocols involving rodents also adhered to the ARRIVE guidelines. All animal study protocols were approved by the Experimental Animal Ethics Committee of Guilin Medical University (Approval No.: GLMU-IACUC-202510134). A total of 86 C^5^7BL/6 J mice (56 males, 18–22 g; 15 females, 16–20 g) were purchased from Guizhou Biouhan Biotechnology Co., Ltd (Guizhou, China) and maintained under specific pathogen-free (SPF) conditions. After 1 week of acclimatization, the rodents were housed in a controlled environment (temperature 22 °C ± 2 °C, relative humidity 60%, 12-h light/dark cycle). At the end of the experimental period, or if severe distress was observed, animals were humanely euthanized in compliance with the AVMA Guidelines for the Euthanasia of Animals (2020 edition). Euthanasia was performed by intraperitoneal injection of an overdose of sodium pentobarbital (150 mg/kg) followed by cervical dislocation to ensure irreversible cessation of cardiac and respiratory activity. The 56 male mice were divided into two sequential experiments with randomized group assignment.

According to the “Chinese Pharmacopoeia”, the clinical dosage of Yuzhu medicinal slices is 9–15 g per day (for an adult weighing 60 kg). After conversion, the dosage per unit body weight is approximately 0.15–0.25 g/kg. The equivalent coefficient for mice is 12.3. Therefore, after conversion, the dosage for mice is approximately 1.85 g/kg to 3.08 g/kg. Based on the results of the pre-experiment and the converted dosages for mice, the low, medium and high doses were 1, 2 and 3 g/kg respectively.

In the first experiment, 36 male mice were allocated to six groups (n = 6 per group). Control mice received weight-adjusted distilled water via oral gavage for three consecutive days, while the model group received equivalent volumes of distilled water. Three treatment groups were administered PO at doses of 1, 2, and 3 g/kg daily for 3 days. The positive control group received N-acetylcysteine (100 mg/kg) for 3 days. Following the second gavage administration, all groups underwent a 16-h overnight fast prior to the third gavage. One hour post-final administration, control animals received intraperitoneal saline (weight-adjusted), while all other groups received APAP (dissolved in warm saline, 300 mg/kg body weight, i. p., dosing volume 10 mL/kg) to induce the experimental model.

The second experiment utilized the remaining male mice (n = 20), divided into seven groups. Control and model groups were treated as above. Pseudo-germ-free (PGF) mice were pretreated with an antibiotic cocktail (vancomycin 100 mg/kg, neomycin sulfate 200 mg/kg, metronidazole 200 mg/kg, and ampicillin 200 mg/kg in sterile water; 200 μL/mouse daily for 7 days) to deplete gut microbiota, followed by maintenance dosing during the 3-day experimental period. Five PGF mice subsequently received 3 g/kg PO for 3 days. All groups followed identical fasting and modeling procedures as in the first experiment.

Female mice (n = 15) underwent procedures identical to those in the first male experiment, except that the treatment duration was extended to 7 days. All other parameters, including dosing regimens and experimental protocols, remained unchanged.

After 24 h of modeling, blood was collected from the eyeball, and mouse liver tissue was collected for subsequent testing (Qin et al., 2025). Serum index evaluation: Mouse eyeball blood was collected, left at room temperature for 1 h, centrifuged at 3000 rpm/min at 4 °C for 15 min, and serum was collected. Serum biochemical indicators such as AST, ALT and LDH are measured according to the kit instructions.

### 2.3 Oxidative stress index detection

Tissue Homogenization and Oxidative Stress Analysis Collected tissue samples (liver and colon) were processed to prepare a 10% (w/v) homogenate. To evaluate oxidative stress, the levels of superoxide dismutase *(SOD),* malondialdehyde *(MDA), and* glutathione (GSH) were quantified in the tissue homogenates and cell lysates. Preliminary pilot experiments were performed to determine the optimal sample dilution ratios. All quantitative assays were conducted strictly in accordance with the manufacturer’s protocols for the respective commercial detection kits.

### 2.4 16S rRNA gene amplicon sequencing

After different interventions on mice, fecal samples were collected before euthanization (The collection of feces ended 24 h after the administration of APAP.) Illumina NovaSeq 6000 sequencing platform was used, and paired-end sequencing was employed to generate 250 bp (PE250) reads from the 5‘and 3′ends of each sequence. To eliminate sequencing errors, raw paired-end (PE) reads were quality-filtered to yield clean data. Overlapping PE reads were then assembled into tags and further screened to isolate target fragments (clean tags). These tags were clustered into Operational Taxonomic Units (OTUs) based on a defined similarity threshold. Finally, taxonomic annotation was performed on the OTUs to determine the microbial community composition of each sample for downstream analysis.

### 2.5 Western blot detection of changes in protein expression levels

Total protein was extracted using RIPA lysis buffer according to the manufacturer’s instructions, and protein concentrations were quantified using the BCA assay. To prepare samples for Western blot analysis, protein concentrations across samples were normalized using a 2% sodium dodecyl sulfate (SDS) solution. The protein solutions were then mixed with an appropriate volume of loading buffer, homogenized by vortexing, and centrifuged briefly. The mixtures were heated at 100 °C for 5 min to denature the proteins and subsequently stored at −20 °C or −80 °C for future use. The proteins were stacked and separated by 4% and 10% SDS polyacrylamide gel electrophoresis (PAGE). Under a constant current of 210mA, the protein was transferred to a polyvinylidene fluoride membrane, sealed with 5% skim milk powder for 2 h, and incubated with primary antibody at 4 °C. The target protein for detection is mouse GAPDH protein as an internal reference. Following primary antibody incubation, the membranes were washed and incubated with the corresponding secondary antibody at room temperature for 1 h. After an additional washing step, protein bands were visualized using enhanced chemiluminescence (ECL) reagents. Immunoblots were captured using an imaging system, and densitometric semi-quantitative analysis of the protein bands was performed using grayscale evaluation.

### 2.6 Mass spectrometry detection

Samples were analyzed using an electrospray ionization (ESI) source monitoring both positive and negative ions (ESI+/ESI−). The instrument was operated in data-dependent acquisition mode, combining a full mass spectrometry scan with secondary tandem MS scanning (Full MS/dd-MS2). The main parameters of the source area were set as follows: the sheath gas flow rate was set to nine arb, the spray voltage was set to 3 kV, the capillary tube (ion transfer tube) temperature was set to 320 °C, the S-lens voltage was set to 55 kV, and the auxiliary gas heating temperature was set to 30 °C. The first-level scanning mode of mass spectrometry was set to Full MS, with a scanning range (m/z) of 100–1,200 and a first-level resolution of 70,000. The number of ions entering the C-Trap was set to 1e6, and the maximum ion injection time was set to 100 m. The second-level scanning mode was set to ddMS2, with a second-level resolution of 35,000 and a vertex excitation time of 3–9 s. The step collision energies were set to 20 kV, 40 kV, and 60 kV, and the dynamic exclusion time was set to 8 s. The chromatographic conditions were as follows: flow rate, 0.4 mL/min; column temperature, 40 °C; injection volume, 4 μL.The PO active ingredient was identified by comparing the output results with the self-built library.

### 2.7 Cell culture

A normal mouse liver cell line,AML-12 (alpha mouse liver 12, CC9038, from Guangzhou Cellcook Biotech Co.,Ltd.). The cells were cultured in DMEM/F-12 medium (Gibco, China) containing 10% (v/v) fetal bovine serum (FBS, FS201-02, TransGen Biotech, China), 1% insulin-transferrin-selenium (ITS, Sigma-Aldrich), and 40 ng/mL dexamethasone (DEX, D4902, Sigma-Aldrich). The cultivation temperature was set to 37 °C. AML-12 cells were pretreated with different concentrations of APAP or RSL3 for 16 h in a humid environment containing 5% carbon dioxide.

### 2.8 Cellular immunofluorescence

Once the cell density reached 70%–80%, the cells were seeded into a 24-well plate and subjected to various treatments. The cell supernatant was discarded, and the cells were fixed in the fixative for 30 min. After membrane rupture and sealing, the primary antibody was incubated at 4 °C overnight. After washing with PBS, the Cy3 secondary antibody was incubated at room temperature for 1 h. The nuclei were stained with DAPI, the slides were sealed, and fluorescence microscopy was used for imaging.

### 2.9 Transcriptome sequencing

For transcriptome sequencing, the Control, Model, and 3 g/kg PO groups were selected, with four samples per group. After detecting the concentration and purity of the extracted RNA, a library was constructed and subjected to quality inspection. The mixed library was gradually diluted and quantified before PE150-mode sequencing on an Illumina sequencer. After data quality control, sequence alignment to the reference genome, and gene expression analysis, differential expression analysis between two comparison combinations was performed using DESeq (v1.38.3) software. Differential analysis of gene expression was performed using DESeq, and the conditions for screening differentially expressed genes were: expression difference fold | log2FoldChange |>1, significant *P*-value < 0.05. GO analysis was performed using topGO (v2.50.0). P-values were calculated using the hypergeometric distribution method, with a P-value of less than 0.05 considered statistically significant for enrichment. GO terms that were significantly enriched with differentially expressed genes (all, upregulated, or downregulated) were identified, and the main biological functions performed by the differentially expressed genes were determined. KEGG pathway enrichment analysis was conducted using clusterProfiler (v4.6.0) software, with particular attention given to significantly enriched pathways exhibiting P-values below 0.05. Gene set enrichment analysis (GSEA) was carried out using GSEA software (v4.1.0). This method did not require the specification of a clear threshold for differentially expressed genes. Instead, all genes were sorted according to their degree of differential expression between the two sample sets, and statistical tests were applied to determine whether a predefined gene set was enriched at either the top or the bottom of the ranked list. The above transcriptome bioinformatics analysis was conducted using the Paisenno Gene Cloud platform (https://www.genescloud.cn).

### 2.10 Statistical analysis

Data from each experimental group were analyzed using GraphPad Prism 8.0, and results were expressed as Mean ± SD. One-way ANOVA was used to compare differences between groups. Tukey’s *post hoc* test was used for data with homogeneous variance, while the Welch and Brown-Forsythe ANOVA test and the Tamhane T2 *post hoc* test were used for data with heterogeneous variance. A *P*-value < 0.05 was considered statistically significant.

## 3 Results

### 3.1 PO attenuates APAP-induced acute liver injury in mice

**FIGURE 1:**
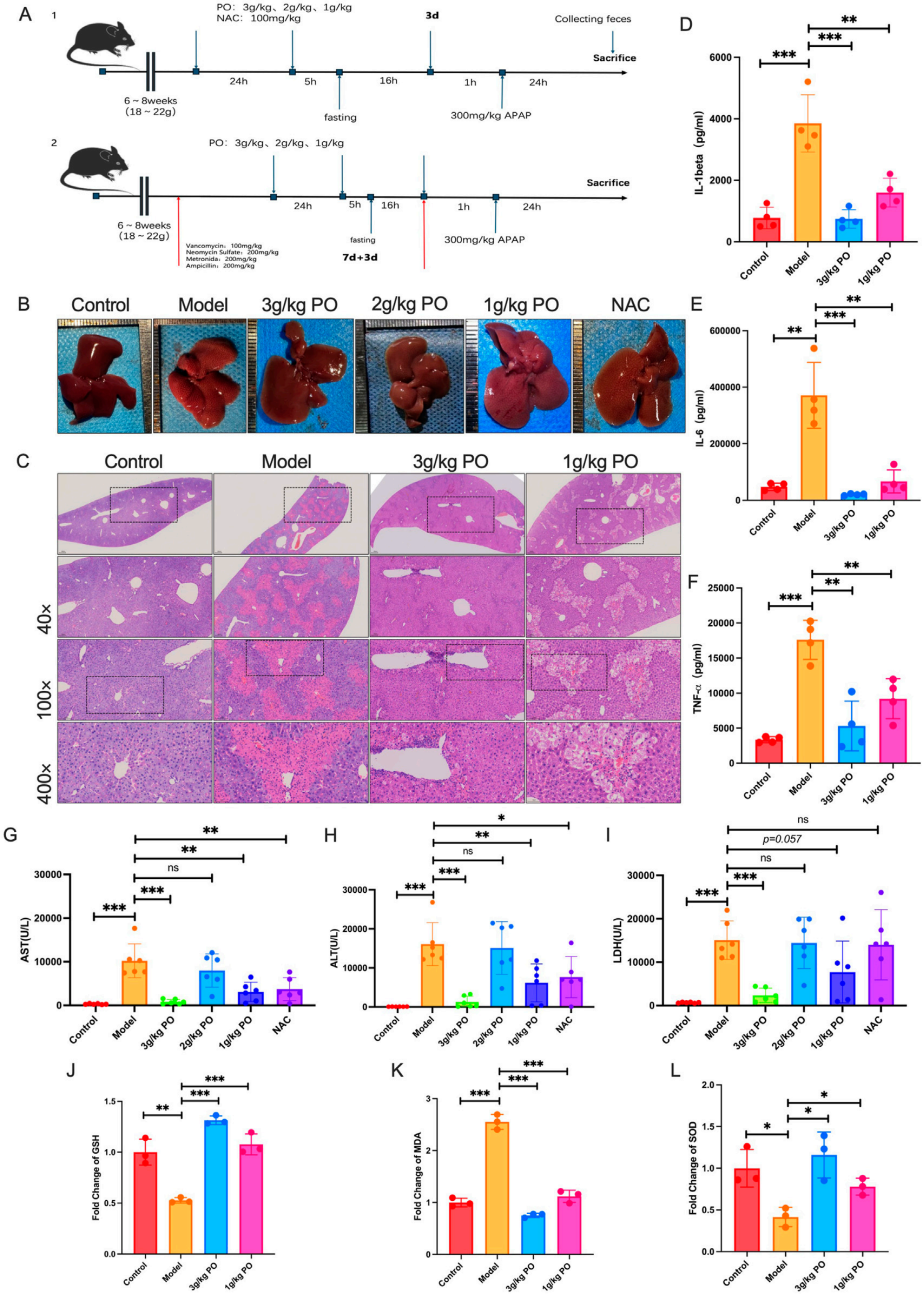
PO alleviates acute liver injury induced by APAP in mice. **(A)** Process flow chart for different groups of mice. **(B)** Appearance of mouse liver. **(C)** H&E staining of mouse liver tissue. **(D–F)** The levels of inflammatory cytokines IL-6, TNF-α, and IL-1beta in liver homogenates of different groups of mice. **(G–I)** Serum AST, ALT, and LDH levels in different groups in different groups. **(J–L)** Content of GSH, MDA, and SOD in liver homogenates of different groups of mice. One point represents a duplicate of a biological sample; *P < 0.05, * *P < 0.01, * * *P < 0.001 indicate statistically significant differences between groups; Ns shows no significant difference.

We first investigated the hepatoprotective effects of PO in a murine model. Upon macroscopic examination, significantly enlarged livers with uneven pale-red discoloration, punctuated hemorrhages, and necrotic lesions accompanied by numerous millet-like white spots were observed in the Model group (Figure 1A). In contrast, these pathological manifestations were markedly ameliorated in both the 3 g/kg PO and 1 g/kg PO treatment groups (Figure 1B). Compared to the Control group, the Model group demonstrated significantly elevated serum levels of liver injury markers (ALT, AST, and LDH), confirming successful establishment of DILI (Figures 1G–I) (Ge et al., 2023). Notably, administration of 3 g/kg PO significantly reduced ALT, AST, and LDH levels compared with the Model group. The protective effect of NAC was comparable only to that of 1 g/kg PO (Figures 1G–I).

To further evaluate hepatic histopathological changes, tissue sections were prepared and stained with H&E. Histological analysis of the Control group showed intact hepatic architecture, characterized by radially arranged hepatocytes around the central vein, with well-defined hepatic cords and normal sinusoids, devoid of necrosis, degeneration, inflammatory infiltration, or fibrosis. In contrast, the Model group exhibited pronounced sinusoidal dilation with congestion, extensive inflammatory cell infiltration, and multifocal hepatocyte eosinophilic degeneration. Bridging necrosis was observed predominantly around the central vein, accompanied by apoptotic bodies (evidenced by chromatin condensation and cell nuclear pyknosis, karyolysis, and cell disruption), vacuolization, and bilirubin-like pigment deposition. Treatment with 3 g/kg PO substantially reversed these pathological alterations, restoring hepatic cord alignment and reducing sinusoidal congestion, with only minor necrotic foci and inflammatory infiltration. Although the 1 g/kg PO group exhibited scattered lesional areas, these were primarily limited to hepatocyte ballooning and edema, with a marked reduction in necrosis compared to the Model group (Figure 1C).

Subsequent investigations focused on the Control, Model, 3 g/kg PO, and 1 g/kg PO groups. The 1 g/kg PO group exhibited lower AST, ALT, and LDH levels than the 2 g/kg group. Given that PO is a complex mixture with potentially non-linear dose-response relationships, this intermediate dose was not included in further phenotypic and mechanistic analyses. ELISA and oxidative stress assays revealed significantly elevated pro-inflammatory cytokines (IL-1beta, IL-6, TNF-α) in the Model group, whereas both PO-treated groups exhibited dose-dependent reductions in these markers (Figures 1D–F). Furthermore, key oxidative stress indicators (SOD, MDA, GSH) were assessed (Figures 1J–L). The Model group exhibited significantly reduced GSH and SOD levels, along with elevated MDA, indicating severe oxidative damage. Conversely, PO administration restored GSH and SOD levels while reducing MDA levels in a dose-dependent manner (Figures 1J–L) (Rezzani and Franco, 2021; Wang et al., 2023). Collectively, these findings demonstrate that *Polygonatum odoratum* extract effectively mitigates APAP-induced acute liver injury by attenuating hepatic inflammation and oxidative stress.

### 3.2 Identification of bioactive components in PO

**FIGURE 2:**
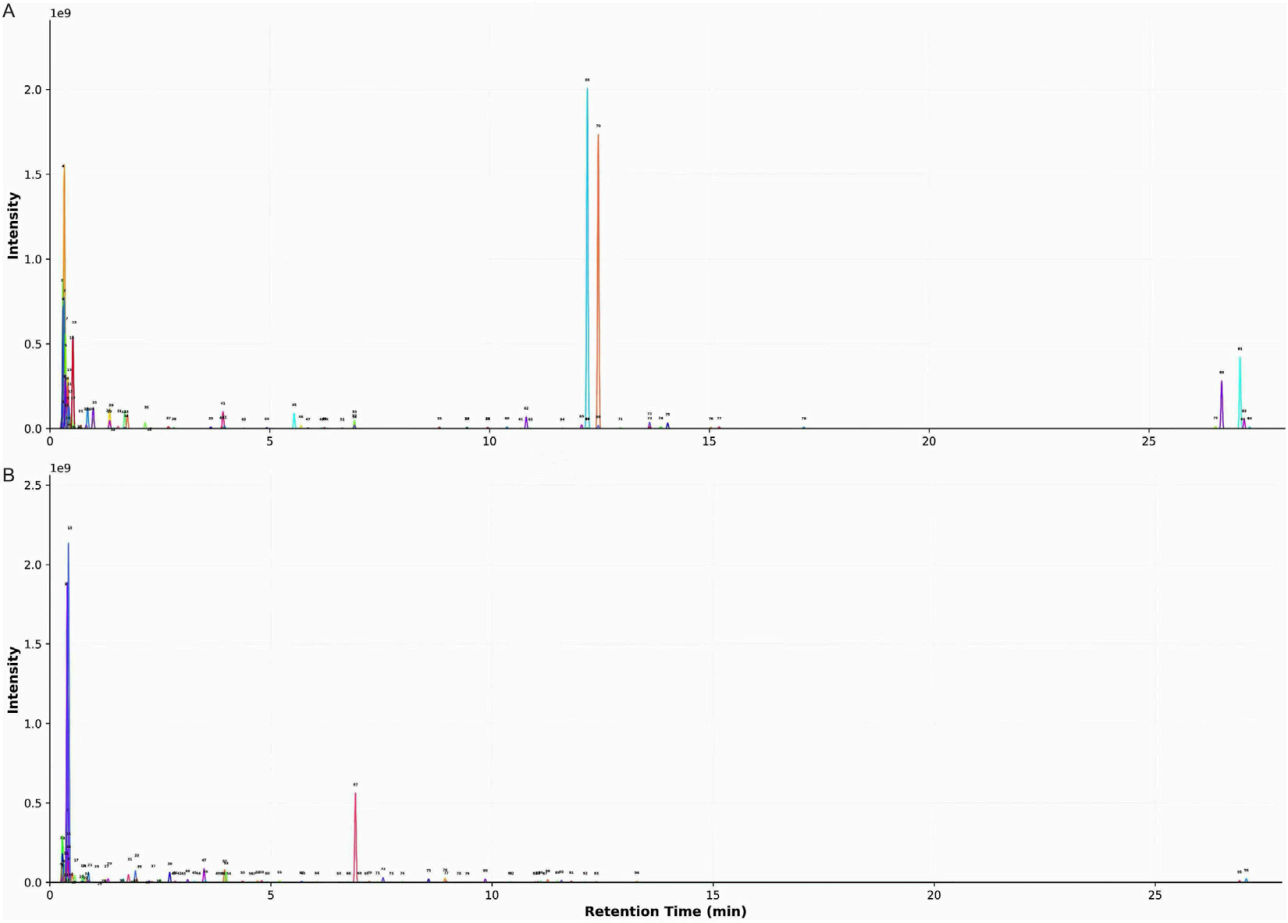
Analysis and identification diagram of PO ultra-high performance liquid chromatography quadrupole time-of-flight mass spectrometry, Figure_2 Analysis and identification diagram of PO ultra-high performance liquid chromatography quadrupole time-of-flight mass spectrometry (UPLC-Q-TOF-MS) **(A)** Negative ion mode chromatogram. **(B)** Positive ion mode chromatogram.

The major bioactive constituents of PO extract were qualitatively analyzed using ultra-performance liquid chromatography coupled with quadrupole time-of-flight mass spectrometry (UPLC-Q-TOF-MS; Figure 2). A self-built compound library search initially identified 170 potential bioactive components (Figure 2). Table 1 presents these 170 compounds (Li et al., 2024).

**TABLE 1:** List of PO compounds identified by UPLC-Q-Orbitrap-MS mass spectrometry.

No.	Index	Name	Formula	Molecular weight	RT_min	Area
1	MPD00018	Uracil	C4H4N2O2	112.03	26.909	11,508,156
2	MPDA00253	N-Acetyl-L-leucine	C8H15NO3	173.11	3.799	1,963,523
3	MPDA00477	2,6-Dihydroxybenzoic acid	C7H6O4	154.03	2.494	17,911,772
4	MPDB01460	Hydroquinidine	C20H26N2O2	326.2	11.795	7,685,950
5	MPD20250615003	DL-isocitric acid lactone	C6H6O6	174.02	0.395	52,178,660
6	MPDA00670	p-Coumaric acid	C9H8O3	164.05	2.712	62,504,087
7	MPDB05251	2-(6-hydroxyhexyl)-3-methylidenebutanedioic acid	C11H18O5	230.12	2.806	2,079,622
8	MPDA00367	Undecanedioic acid	C11H20O4	216.14	6.042	4,113,677
9	MPDA00982	Citric acid	C6H8O7	192.03	0.395	1,887,006,180
10	MPDA00295	(S)-Leucic acid	C6H12O3	132.08	1.315	22,005,118
11	MPDB01325	Traumatic acid	C12H20O4	228.14	6.54	1,991,471
12	MTD20250521002	2-Hydroxypalmitic acid	C16H32O3	272.24	13.277	6,842,703
13	MTD14946	Pyruvic acid	C3H4O3	88.016	0.395	128,576,522
14	MPD00057	1-Aminocyclopropanecarboxylate	C4H7NO2	101.05	0.327	773,711,384
15	MTD05073	Lactic acid	C3H6O3	90.03,169,405	0.422	2,133,281,795
16	MPDB04623	N-trans-p-coumaroyloctopamine	C17H17NO4	299.12	3.521	13,077,763
17	MPDA00238	Ferulic acid	C10H10O4	194.06	3.272	7,343,296
18	MPDB01804	Cyclo (Tyr-pro)	C14H16N2O3	260.12	1.331	8,518,896
19	MPDA00599	α-Cyperone	C15H22O	218.17	10.396	9,626,873
20	MPDA00756	5-Hydroxyindole-3-acetic acid	C10H9NO3	191.06	1.906	13,233,678
21	MPDB02591	[3,4,5-Trihydroxy-6-(hydroxymethyl)oxan-2-yl]3-hydroxy-4,5-dimethoxybenzoate	C15H20O10	360.11	1.03	2,832,780
22	MPD202506220691	3-Furoic acid	C5H4O3	112.016044	0.395	208,788,505
23	MPDC05442	Trehalose	C12H22O11	342.12	0.286	290,860,965
24	MPDB01738	N-Feruloyloctopamine	C18H19NO5	329.13	3.954	79,759,695
25	MPDA00808	Sucrose	C12H22O11	342.12	0.842	38,657,034
26	MTD20055	Erythronolactone	C4H6O4	118.0266,087	0.422	2,4,691,900
27	MPD202506221023	1,2,4-Benzenetriol	C6H6O3	126.0316,941	0.565	43,121,536
28	MTD14420	D-5-oxoproline	C5H7NO3	129.0425,931	0.395	193,273,289
29	MTD20272	3-Hydroxybenzaldehyde	C7H6O2	122.0367,794	1.779	48,497,767
30	MTD05241	MALATE	C4H6O5	134.0215,233	0.368	358,262,584
31	MPDA00628	Hyperoside	C21H20O12	464.1	4.047	872,748
32	MTD14910	Itaconic acid	C5H6O4	130.0266,087	27.06	23,322,134
33	MPDB04141	5-Hydroxy-7-[4-hydroxy-2-methoxy-3-(3-methylbut-2-enyl)phenyl]-2,2-dimethyl-7,8-dihydropyrano [3,2-g]chromen-6-one	C26H28O6	436.19	9.958	5,058,379
34	MPDB04972	{3-[(E)-2-(1,3-Benzodioxol-5-yl)vinyl]-2-oxiranyl}(1-piperidinyl)methanone	C17H19NO4	301.13	0.382	87,345,912
35	MPD202506220744	3,5,2′,4′-tetramethoxystilbene	C18H20O4	300.1,361,591	12.225	5,507,043
36	CNP02711811	Grayanotoxin XVIII	C20H32O4	336.23006	9.487	4,890,089
37	MPD202506220961	1-Naphthalenepentanoic acid, 5-carboxy-1,2,3,4,4a,7,8,8a-octahydro-7-hydroxy-beta,1,2,4a-tetramethyl-	C20H32O5	352.2,249,741	6.928	16,897,865
38	MPDB03029	Acanthoside B	C28H36O13	580.22	4.546	1,439,158
39	MPDA00972	D-erythro-dihydrosphingosine	C18H39NO2	301.3	10.836	69,154,377
40	MTD15267	3-Phenyllactic acid	C9H10O3	166.0629,942	1.936	72,486,793
41	MPDA00742	Pipecolic acid	C6H11NO2	129.08	0.354	556,909,522
42	MTD17121	[(1S,6S,7R)-6-acetyloxy-1-(3-methylbutanoyloxy)spiro [4a,5,6,7a-tetrahydro-1h-cyclopenta [c]pyran-7.2′-oxirane]-4-yl]methyl 3-methylbutanoate	C22H32O8	424.209,718	13.641	9,843,915
43	MPDA00623	Naringenin	C15H12O5	272.07	6.758	2,586,110
44	MPDA00213	Betaine	C5H11NO2	117.08	26.656	280,159,000
45	MTD05274	Mannitol	C6H14O6	182.0790,382	0.286	36,774,438
46	CNP04917750	6-Acetamidohexanoic acid	C8H15NO3	173.10519	1.157	2,887,473
47	MPD202506220312	Cresopirine	C10H10O4	194.0579,088	8.969	5,785,442
48	MPD10457	Octanedioic acid	C8H14O4	174.0892,089	2.245	10,642,874
49	CNP02318841	(2 S)-2-isopropylmalate	C7H12O5	176.06847	0.874	11,934,227
50	MTD21156	Gluconic acid	C6H12O7	196.0583,027	0.314	120,419,232
51	MPDB02167	Homoeriodictyol	C16H14O6	302.08	7.005	3,650,564
52	MTD15270	Indolelactic acid	C11H11NO3	205.0738,932	2.932	2,387,813
53	MTD16155	Hydroxysebacic acid	C10H18O5	218.1,154,237	2.836	7,865,099
54	MPDA00225	Maltotriose	C18H32O16	504.17	0.286	181,115,474
55	MTD12734	3-Methoxytyrosine	C10H13NO4	211.0844,579	1.658	22,014,753
56	MTD20978	Dodecanedioic acid	C12H22O4	230.1,518,092	7.162	1,895,776
57	CNP01396471	PyroGlu-val	C10H16N2O4	228.11101	0.75	7,206,635
58	MPDB02997	Isorhamnetin 3-O-glucosyl-6‘’-rhamnosid	C28H32O16	624.17	4.609	1,048,251
59	MPDB02559	Gentisic acid 5-O-beta-glucoside	C13H16O9	316.08	0.719	8,496,245
60	MTD16140	Enoic acid	C18H34O5	330.241	6.913	561,343,702
61	MTD18534	Oroxylin A	C16H12O5	284.0684,735	27.138	3,816,212
62	MTD13447	Erucamide	C22H43NO	337.334,465	13.895	1,0,371,860
63	MTD16016	Beta-hydroxymyristic acid	C14H28O3	244.2,038,448	11.039	1,924,669
64	MTD10718	9-Hode	C18H32O3	296.2,351,449	11.039	5,910,065
65	MTD06621	PHOSPHORYLCHOLINE	C5H14NO4P	183.0660,446	12.474	17,848,746
66	MTD12667	4-Hydroxy-2′,3.4′,6′-tetramethoxychalcone	C19H20O6	344.1,259,884	11.197	1,528,573
67	MPD20250615004	Chelidonic acid	C7H4O6	184	0.507	60,180,917
68	MPDA00228	Sebacic acid	C10H18O4	202.12	4.793	10,760,328
69	MTD10111	Zearalenone	C18H22O5	318.1,467,238	9.44	1,444,100
70	MTD16166	(10e,15z)-9,12,13-trihydroxyoctadeca-10,15-dienoic acid 95,341–44-9	C18H32O5	328.2,249,741	7.538	29,468,687
71	MTD21180	Maltotetraose	C24H42O21	666.2,218,584	0.286	125,873,544
72	MTD15084	Fructoselysine	C12H24N2O7	308.1,583,511	0.258	4,942,746
73	CNP05578490	Cocamidopropyl betaine	C19H38N2O3	342.28824	8.86	8,012,927
74	MTD20728	Diferuloyl putrescine	C24H28N2O6	440.195	5.698	7,120,574
75	MTD15739	N-Fructosyl pyroglutamate	C11H17NO8	291.0954,165	0.395	57,221,524
76	MTD11055	Hydroxystearic acid	C18H36O3	300.266,445	12.363	3,146,128
77	MTD16167	9-Hydroxy-10,12-octadecadienoic acid	C18H32O3	296.235	11.26	17,097,052
78	MTD12660	Feruloyl dehydrotyramine	C18H17NO4	311.115,758	3.939	98,416,056
79	MTD11126	Arachidoyl ethanolamide	C22H45NO2	355.3,450,297	12.977	5,076,126
80	MPD202506220534	5-[(6-Ethoxy-3,4,5-trihydroxyoxan-2-yl)methoxy]-3-hydroxy-3-methyl-5-oxopentanoic acid	C14H24O10	352.136,947	0.75	46,054,791
81	MTD11078	Stearamide	C18H37NO	283.2,875,148	15.034	7,309,983
82	MTD14306	Feruloyl tyramine	C18H19NO4	313.1,314,081	5.557	88,745,639
83	MTD16170	(9Z,12 E)-15,16-dihydroxyoctadeca-9,12-dienoic acid	C18H32O4	312.2,300,595	8.939	24,165,670
84	MTD14372	Paprazine	C17H17NO3	283.1,208,434	5.197	10,128,670
85	MTD15238	N-Fructosyl isoleucine	C12H23NO7	293.1,474,521	0.409	171,647,977
86	MTD20250526090	N,N′-dicoumaroylputrescine	C22H24N2O4	380.1,736,073	5.87	3,785,434
87	MTD11090	Methyl stearate	C19H38O2	298.2,871,805	11.654	2,834,111
88	MPD20849086	Succinoadenosine	C14H17N5O8	383.1,077,125	0.671	12,155,825
89	MPD202506220122	N-Acetylvanilalanine	C12H15NO5	253.0950,226	0.828	21,824,574
90	CNP02439250	Palmitamide	C16H33NO	255.25621	13.641	36,082,269
91	CNP01915630	9-Oxooctadeca-10,12-dienoic acid	C18H30O3	294.21949	6.928	48,403,784
92	MTD15240	N-Fructosyl phenylalanine	C15H21NO7	327.131,802	0.493	88,402,130
93	CNP04529010	9,12-Octadecadiynoic acid	C18H28O2	276.20893	6.928	27,045,397
94	MPD202509211439	2-Benzofurancarboxylic acid, methyl ester	C10H8O3	176.0473,441	3.97	15,787,487
95	MTD20160	Sakuranetin	C16H14O5	286.084	4.918	2,794,558
96	NPC65530	Piceatannol 3′-O-glucoside	C20H22O9	406.1,263,823	3.924	2,547,852
97	MTD14610	[6]Gingerol	C17H26O4	294.1,831,093	8.567	20,742,020
98	MPD20250714084	12 (13)-epoxy-9z-octadecenoic acid	C18H32O3	296.2,351,449	9.487	8,265,186
99	MPD202506220540	5,9,13-Pentadecatrien-2-one, 6,10,14-trimethyl-	C18H30O	262.2,296,656	10.93	2,553,816
100	CNP05287801	H-ILE-LEU-OH	C12H24N2O3	244.17869	1.455	2,926,012
101	MPD20250714009	Gly-tyr	C11H14N2O4	238.0953,569	0.466	33,352,955
102	MPDB05310	3,15-Dihydroxyhexadecanoic acid	C16H32O4	288.23	7.227	7,820,287
103	MPDB05131	Auraptene	C19H22O3	298.16	10.412	3,685,493
104	MPD202509211257	Terrestribisamide	C24H28N2O6	440.1,947,366	5.713	18,310,791
105	MPDA00955	L-Tryptophan	C11H12N2O2	204.09	0.984	62,713,521
106	MPDB05319	(E)-Undec-2-enedioic acid	C11H18O4	214.12	5.729	3,902,785
107	MPD20250714060	PyroGlu-trp	C16H17N3O4	315.121,906	3.025	1,300,254
108	MTD16290	Phthalic anhydride	C8H4O3	148.016044	12.225	2,005,464,694
109	CNP02767100	Coriandrin	C13H10O4	230.05791	27.291	8,786,029
110	MTD20250408031	PyroGlu-phe	C14H16N2O4	276.111,007	2.291	4,009,924
111	MTD20222	Quercetin-3-O-rutinoside	C27H30O16	610.1,533,849	3.893	963,867
112	MTD14381	Dicoumaroyl spermidine	C25H31N3O4	437.231	3.985	67,446,223
113	MTD22539	(2S,3R,4R,5R,6S)-2-{[(2R,3S,4R,5R,6S)-6-{[(2R,3R,4S,5S,6R)-4,5-dihydroxy-6-(hydroxymethyl)-2-[(1′S,2 S,4′S,5 S,7′R,9′S,13′R,16′S)-5.7′,9′,13′-tetramethyl-5-({[(2R,3R,4S,5S,6R)-3,4,5-trihydroxy-6-(hydroxymethyl)oxan-2-yl]oxy}methyl)-5′-oxaspiro [oxolane-2.6′-pentacyclo [10.8.0.0??0??0????]icosan]-18′-eneoxy]oxan-3-yl]oxy}-4,5-dihydroxy-2-(hydroxymethyl)oxan-3-yl]oxy}-6-methyloxane-3,4,5-triol	C51H82O23	1,062.524,689	7.413	4,233,492
114	MPD202506220700	3-Aminocoumarin	C9H7NO2	161.0476,785	1.36	46,000,673
115	MPD202506220448	6-Hydroxy-3,4-dihydro-2(1H)-quinolinone	C9H9NO2	163.0633,285	0.436	127,675,538
116	MTD20250408022	Leu-pro	C11H20N2O3	228.1,473,925	0.704	19,880,743
117	MTD09527	Flindersine	C14H13NO2	227.0946,287	4.407	3,005,425
118	MTD15567	Piperonylic acid	C8H6O4	166.0266,087	12.225	3,553,142
119	MPDB04310	1-O-[(3beta,5xi,9xi,18xi)-3-(beta-D-Glucopyranuronosyloxy)-23-hydroxy-28-oxoolean-12-en-28-yl]-beta-D-glucopyranose	C42H66O15	810.44	7.724	1,548,795
120	MPD202506220111	Norsalsolinol	C9H11NO2	165.0789,786	0.522	536,448,586
121	MTD13473	Guanine	C5H5N5O	151.0494,098	0.436	72,074,652
122	MTD13775	2-Naphthylamine	C10H9N	143.0734,993	1.36	115,362,189
123	MTD21649	p-Hydroxy-cinnamic acid	C9H8O3	164.0473,441	2.696	11,483,140
124	MTD14307	Feruloyl putrescine	C14H20N2O3	264.147	1.548	13,122,691
125	MTD13444	2-Phenylacetamide	C8H9NO	135.0684,139	1.763	8,4,031,831
126	MTD10360	Coniine	C8H17 N	127.1,360,995	26.515	12,014,804
127	MPDA00304	Ricinoleic acid	C18H34O3	298.25	11.573	12,580,263
128	MPD202506220089	Phloroglucinol	C6H6O3	126.0316,941	27.075	419,664,181
129	MPD00147	Caffeine	C8H10N4O2	194.08	1.704	107,051,548
130	MTD07884	Indoline	C8H9N	119.0734,993	0.522	544,559,842
131	MPDB05288	4-Oxododecanedioic acid	C12H20O5	244.13	4.359	7,617,402
132	MPDB02490	2-(beta-D-Glucopyranosyloxy)benzyl (2 E)-3-(3,4-dihydroxyphenyl)acrylate	C22H24O10	448.14	9.25	1,684,035
133	MPDA00324	Gallic acid	C7H6O5	170.02	0.565	9,263,300
134	MPD00141	Pantothenic acid	C9H17NO5	219.11	0.551	18,577,794
135	MPDA01103	3-Hydroxybenzoic acid	C7H6O3	138.03	1.253	19,110,003
136	MPDA00930	Salicylic acid	C7H6O3	138.03	3.117	16,925,384
137	MTD20250526107	Sinapic acid O-glucoside	C17H22O10	386.12	1.997	2,135,520
138	MPDC05526	LPC(16:0/0:0)	C24H50NO7P	495.33	12.474	1,734,620,058
139	MPDB04624	N-trans-feruloyltyramine	C18H19NO4	313.13	4.7	9,869,741
140	MPDB04694	Cyclo (Phe-pro)	C14H16N2O2	244.12	2.821	5,006,933
141	MPDB01660	Atractylenolide III	C15H20O3	248.14	6.181	2,955,008
142	MPDB05305	9-(2,3-dihydroxypropoxy)-9-oxononanoicacid	C12H22O6	262.14	3.367	2,814,879
143	MPDA00836	Nicotinic acid	C6H5NO2	123.03	0.874	63,340,918
144	MPDB01917	(2 E)-5-(2,3-Dimethyltricyclo [2.2.1.0–2.6∼]hept-3-yl)-2-methyl-2-pentenoic acid	C15H22O2	234.16	11.479	7,600,232
145	MPDA00474	Oleamide	C18H35NO	281.27	14.053	31,965,899
146	MTD21731	Verbasoside	C20H30O12	462.1,737,264	6.243	6,776,310
147	MPD20250615005	3-Hydroxy-3-methyl-5-oxo-5-[[(2R,3S,4S,5R,6R)-3,4,5-trihydroxy-6-propan-2-yloxyoxan-2-yl]methoxy]pentanoic acid	C15H26O10	366.15	1.253	4,897,895
148	MPDA00510	Caffeic acid	C9H8O4	180.04	1.966	23,653,942
149	MPDA00244	Sinomenine	C19H23NO4	329.16	1.704	8,363,436
150	MPDA00780	PHYTOSPHINGOSINE	C18H39NO3	317.29	10.711	3,392,789
151	MTD11184	Dodecyl gallate	C19H30O5	338.2,093,241	12.112	2,675,720
152	MPDA00709	Emodin	C15H10O5	270.05	10.444	2,818,632
153	MPDA00964	L-Arginine	C6H14N4O2	174.11	0.301	883,212,140
154	MTD20093	Triptophenolide	C20H24O3	312.1,725,446	11.103	9,104,248
155	MPDA00459	sn-Glycero-3-phosphocholine	C8H20NO6P	257.1	0.327	1,556,713,511
156	CNP01175652	MENTHYL SALICYLATE	C17H24O3	276.17254	10.977	3,039,773
157	MPDA00676	Hexadecanedioic acid	C16H30O4	286.21	7.972	3,183,712
158	MPDB02172	Naringenin5-methylether	C16H14O5	286.08	4.933	5,620,921
159	MPDB01600	Dihydrocapsaicin	C18H29NO3	307.21	6.274	3,655,197
160	MPD00027	Agmatine	C5H14N4	130.12	0.272	67,314,307
161	MPDA00771	Isonicotinic acid	C6H5NO2	123.03	0.857	125,295,368
162	MPDA01102	2-Picolinic acid	C6H5NO2	123.03	27.168	53,615,941
163	MPDA01037	3-Indoleacetic acid	C10H9NO2	175.06	3.659	8,839,850
164	MPDA00270	Azelaic acid	C9H16O4	188.1	3.49	85,326,645
165	MPDA00841	L-Tyrosine	C9H11NO3	181.07	0.409	146,699,639
166	MPDA00729	2-(1H-indol-3-yl)ethan-1-ol	C10H11NO	161.08	3.909	13,015,560
167	MPDA00321	L-Proline	C5H9NO2	115.06	0.327	400,287,729
168	MPDA00956	Adenine	C5H5N5	135.05	0.354	316,036,415
169	MPDA00959	L-isoleucine	C6H13NO2	131.09	0.409	304,533,517
170	MPDB02392	1-Naphthalenecarboxylic acid,5-[2-(2,5-dihydro-2-oxo-3-furanyl)ethyl]decahydro-1,4a-dimethyl-6-methylene-,methylester	C21H30O4	346.21	6.649	2,909,947

### 3.3 Active targets and enriched pathways of PO in preventing APAP-induced acute liver injury

To identify the bioactive components of PO extract, we conducted a comprehensive search of multiple databases, including TCMSP. Using stringent criteria of oral bioavailability (OB) > 30% and drug-likeness (DL) > 0.18, we identified eight major bioactive compounds (Supplementary Table S1) (Luo et al., 2023). The SMILES structures of these compounds were retrieved from PubChem and subsequently analyzed using the Swiss Target Prediction database. After removing duplicate entries, 265 potential targets were identified (Supplementary Figure S1A) (Shang et al., 2023).

Next, we cross-referenced these PO-related targets with known acute liver injury-related targets, confirming the initial 265 potential targets. A protein-protein interaction (Mavilavalappil et al., 2026) network, disease component target map, and disease component target pathway map were constructed using Cytoscape to further elucidate the key genes involved in the hepatoprotective effect of PO on ALI (Supplementary Figures S1B-D) (Murtaza et al., 2022). To characterize these potential targets, we conducted Gene Ontology (GO) analysis and KEGG pathway enrichment analysis (Supplementary Figures S1E-H). Notably, KEGG pathway analysis indicated significant enrichment of PO targets in the sphingolipid signaling pathway, suggesting its potential regulatory role in APAP induced liver injury (Supplementary Figures S1E-HA).

Interestingly, among the eight active components selected by TCMSP, we did not detect them in the UPLC-Q-TOF-MS identification and detection process. Therefore, the pathways and targets predicted by network pharmacology may differ from the actual ones. We only use the network pharmacology results as an exploratory analysis.

### 3.4 PO modulates gut microbiota to ameliorate APAP-induced acute liver injury

**FIGURE 3:**
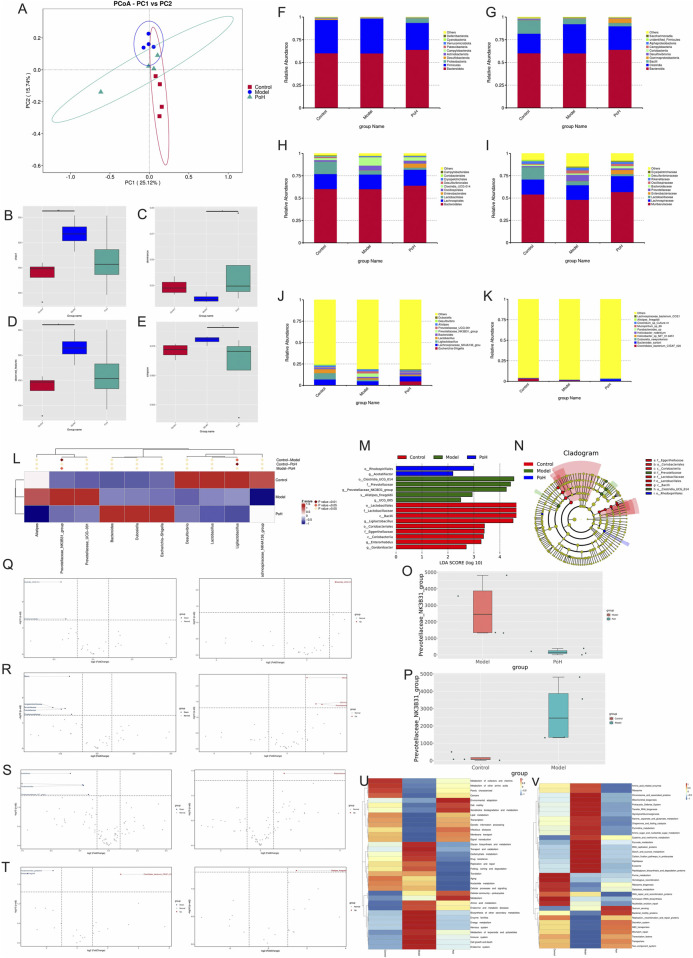
16S rDNA reveals the mechanism of PO regulation of gut microbiota. **(A)** PCoA analysis of beta diversity changes between groups. **(B–E)** Chao1, dominance, observed features, Simpson diversity index analysis. Column chart of relative abundance of the top 10 species at the phylum, family, genus, and species (p, c, o, f, g, s) level among each group of samples **(F–K). (L)** Top 10 significant changes in gut microbiota heatmap on a horizontal level. **(M,N)** LEfSe analysis of gut microbiota and evolutionary tree between groups. The relative changes of **(O,P)** Prevotellace-NK3B31 Group in Control vs. Model and Model vs. PoH. Control vs. Model and Model vs. PoH volcano maps at the **(Q–T)** phylum (c, o, f, g) level. **(U,V)** Functional prediction analysis showing modulation of metabolic pathways, including lipid metabolism, across Control, Model, and PoH groups.

Increasing evidence has demonstrated the critical role of the gut-liver axis in drug absorption and metabolism, with gut microbiota dysbiosis strongly associated with various liver pathologies (Cao et al., 2024; Li et al., 2023; Lucchetti et al., 2024). To assess the impact of PO on gut microbial composition, we conducted 16 S rDNA sequencing on fecal samples collected from three experimental groups: Control, APAP-induced Model, and Model treated with 3 g/kg PO (PoH). Alpha-diversity analysis using Chao1 and observed-features indices revealed significantly higher microbial diversity in the Model group than in the Control, whereas the PoH treatment did not alter diversity relative to the Model group (Figures 3B,D). Intriguingly, no significant difference was found between the Model and Control groups by dominance analysis. However, compared to the Model group, a significant increase in dominance was observed following PoH treatment (Figure 3C). Conversely, Simpson index analysis showed no significant variation between Model and Control groups, yet PoH treatment significantly reduced diversity relative to the Model group (Figure 3E).

**FIGURE 4:**
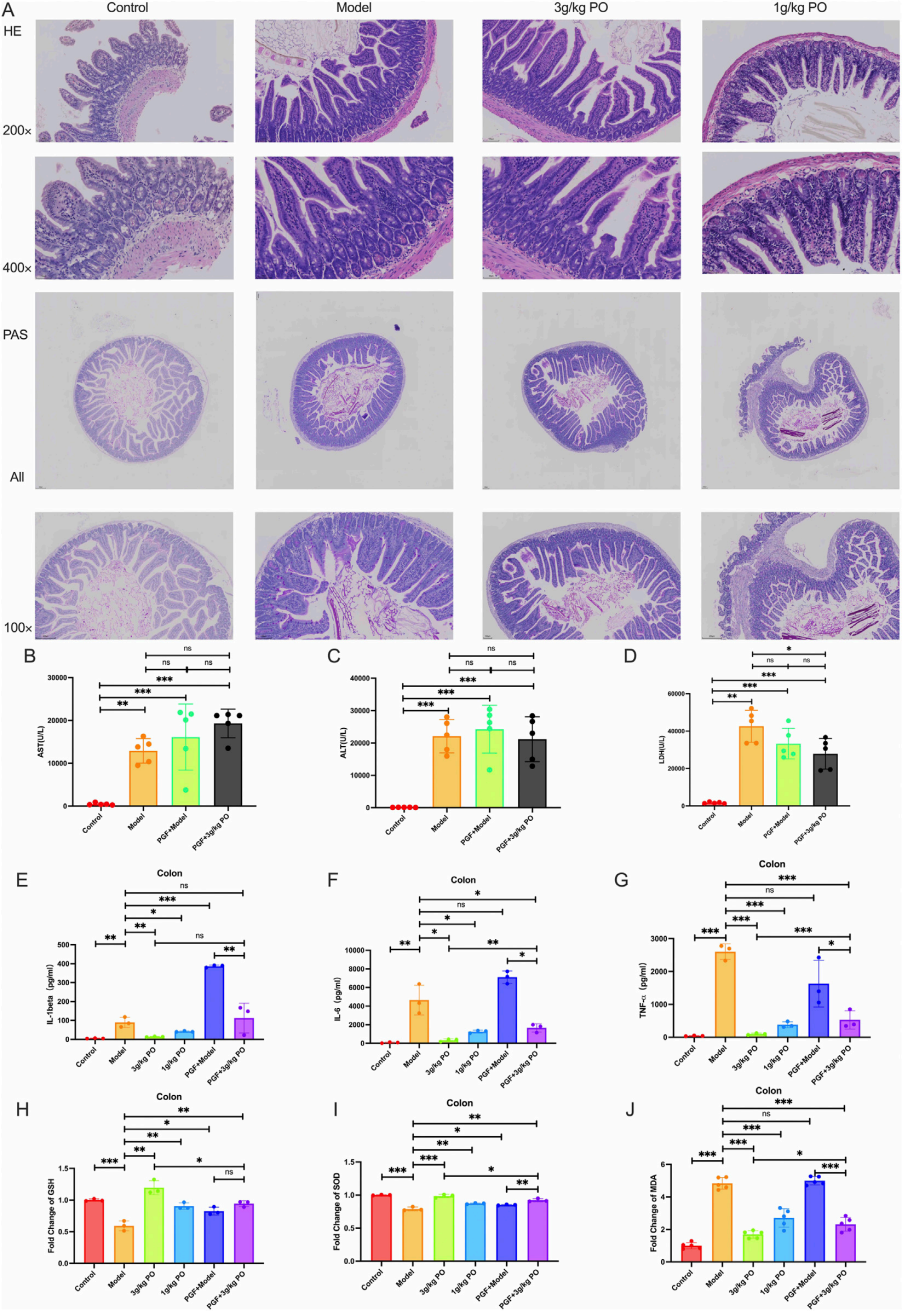
PO improves intestinal outcomes and alleviates inflammation and oxidative stress in colon tissues. **(A)** HE staining and PAS staining of small intestine sections from different experimental groups. **(B–D)** Changes in liver function indicators ALT, AST, and LDH in different groups of mice. **(E–G)** Changes in inflammatory cytokine levels in colon tissues across treatment groups. **(H–J)** Oxidative stress markers (GSH, SOD, MDA) in colon tissues across treatment groups. Each dot represents an independent biological replicate. Statistical significance: *P < 0.05, **P < 0.01, ***P < 0.001 (intergroup comparisons); “ns” indicates no significant difference.

Taxonomic profiling at the phylum (p), class (c), order (o), family (f), genus (g), and species (s) levels demonstrated distinct microbial shifts. At the phylum level, the relative abundance of Proteobacteria and Bacteroidota was elevated by POH while Verrucomicrobiota was suppressed (Figure 3F). The Model group exhibited increased Clostridia at the class level, which was counteracted by PoH in favor of Gammaproteobacteria enrichment (Figure 3G). Order-level analysis revealed Model-associated increases in Clostridia-UCG-014 and Oscillospirales, whereas PoH upregulated Enterobacterales while suppressing these taxa (Figure 4H). Family level alterations included elevated Lactobacillaceae and Prevotellaceae in the Model group, while PoH enhanced Bacteroidaceae and Enterobacteriaceae while reducing Prevotellaceae (Figure 3I). Notably, at the genus level, Prevotellaceae-NK3B31-Group and Prevotellaceae-UCG-001 were enriched in the Model group but significantly diminished by PoH, which concurrently increased Escherichia-Shigella abundance (Figure 3J). Species-level analysis identified *Bacteroides* sartorii as a PoH enriched taxon (Figure 3K).

Further analysis of the differentially abundant microbiota among the Control, Model, and PoH groups revealed significant taxonomic alterations at multiple phylogenetic levels. At the order level, Clostridia_UCG-014 and Christensenellales showed significant reduction in the Model group compared to the Control group, while Clostridia_UCG-014 was significantly elevated in the PoH group relative to the Model group (Figure 3Q). Family level analysis demonstrated notable decreases in Prevotellaceae, Tannerellaceae, Christensenellaceae, Hungateiclostridiaceae, and unclassified taxa (Others) in the Model group versus Control, with Prevotellaceae, UCG-010, and Others being significantly increased in the PoH group compared to Model (Figure 3R). Genus-level comparisons revealed significant declines in Parabacteroides, Christensenellaceae_R-7_group, Acetatifactor, and A2 in the Model group relative to Control, whereas Butyricicoccus was significantly upregulated in the PoH group versus Model (Figure 3S). Species-level analysis indicated that Alistipes_finegoldii and Parabacteroides_goldsteinii were significantly reduced while Clostridiales_bacterium_CIEAF_012 was markedly increased in the Model group compared to Control. In contrast, Alistipes_finegoldii showed significant elevation in the PoH group relative to Model. Collectively, these findings demonstrated that APAP modeling resulted in a decrease in the abundance of most affected bacterial taxa, with Clostridiales_bacterium_CIEAF_012 as the sole exception, showing increased abundance (Figure 3T). Notably, all significantly altered microbiota regulated by 3-day PoH pretreatment exhibited increased abundance compared to the Model group (Figures 3L,Q–T).

LEfSe analysis further delineated group-specific microbial signatures: Lactobacillales and related taxa (e.g., Lactobacillaceae) were enriched in Controls (high LDA scores), while Clostridia_UCG-014 and Prevotellaceae characterized the Model group (Figure 3M). PoH-associated biomarkers included Rhodospirillales and Acetobacteraceae. Phylogenetic analysis of 16 S rDNA sequences confirmed distinct clustering among groups, with Eggerthellaceae (Controls), Prevotellaceae (Model), and Rhodospirillales (PoH) serving as key discriminative taxa (Figure 3N).

Collectively, these findings demonstrate that PO restores gut microbial homeostasis, attenuates dysbiosis. Functional prediction suggests that PO may exert hepatoprotective effects via modulation of lipid metabolism pathways (Figures 3U,V) (Cao et al., 2024; Gao, 2024).

### 3.5 PO alleviates APAP-induced intestinal stress and inflammation and regulate the gut microbiota

Consistent with 16 S rDNA sequencing results demonstrating PO-mediated modulation of gut microbiota, histological examination through H&E and PAS staining revealed significant intestinal morphological alterations in the Model group. These pathological changes appeared to be ameliorated by PO pretreatment (Figure 4A) (Esteban-Torres et al., 2023).

To further investigate the role of the gut microbiota, pseudo-germ-free (PGF) mice were established by administering a quadruple antibiotic cocktail for 1 week (Figure 1A2). Subsequent experimental treatments included either PO pretreatment followed by APAP challenge (PGF+3 g/kg PO) or APAP challenge alone (PGF + Model). Biochemical analysis demonstrated that antibiotic-mediated depletion of the gut microbiota neither exacerbated nor attenuated APAP-induced hepatotoxicity, as reflected by comparable AST, ALT and LDH levels between the Model and PGF + Model groups. Notably, the hepatoprotective effects of PO were completely abolished in PGF mice (Figures 4B–D) (Wu et al., 2025).

Parallel examinations of colonic tissues revealed that APAP treatment significantly elevated pro-inflammatory cytokines (IL-6, TNF-α, and IL-1beta) in both conventional and PGF mice, with IL-1beta showing a particularly pronounced increase in the PGF + Model group compared to the Model group. Importantly, 3 g/kg PO pretreatment substantially mitigated these inflammatory responses in PGF mice (Figures 4E–G). Oxidative stress parameters showed concordant patterns: both the Model and PGF + Model groups exhibited marked reductions in GSH and SOD activities, along with elevated MDA levels, compared to controls. While PO pretreatment partially restored these oxidative markers in conventional mice, its efficacy was significantly attenuated in PGF mice (Figures 4H–J). Collectively, these findings demonstrate that APAP induces both inflammatory responses and oxidative stress in colonic tissues. PO effectively alleviates these pathological processes primarily through mechanisms involving modulation of the gut microbiota.

### 3.6 PO modulates ceramide metabolism and attenuates hepatic ferroptosis

**FIGURE 5:**
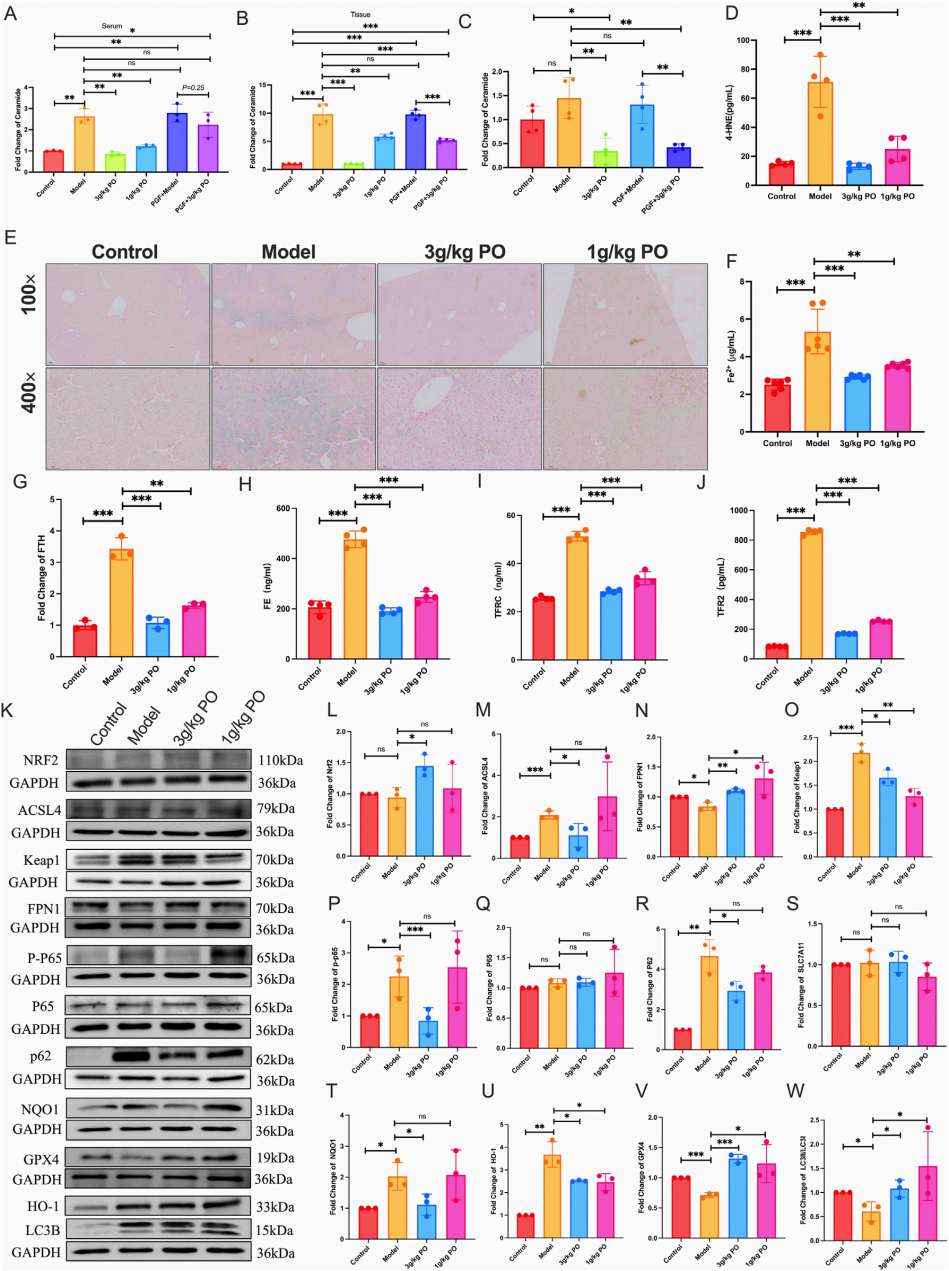
PO can regulate ceramide content, reduce ferroptosis and regulate autophagy and inflammation. **(A)** Changes in serum ceramide levels in mice. **(B)** Changes in ceramide content in mouse liver tissue homogenate. **(C)** Changes in ceramide content in mouse colon tissue homogenate. **(D)** Changes in 4-HNE content in mouse liver tissue homogenate. **(E)** Representative image of Prussian blue staining of mouse liver. **(F)** Changes in ferrous ion content in mouse liver tissue homogenate. **(G)** Changes in the content of iron heavy chain protein FTH in mouse liver tissue. **(H)** Changes in ferritin FE content in mouse liver tissue. **(I)** Changes in TFR content in mouse liver tissue. **(J)** Changes in TFR2 content in mouse liver tissue. **(K)** Representative Western blot image. **(L–W)** Western blot: Relative quantitative analysis of NRF2, ACSL4, FPN1, KEAP1, p-P65, P65, P62, HO-1, NQO1, GPX4, LC3B. Each dot represents a biological replicate. Statistical significance: *P < 0.05, **P < 0.01, ***P < 0.001 (compared between groups); “ns” indicates no significant difference.

Network pharmacology analysis identified the sphingolipid signaling pathway as one of the key mechanisms underlying PO-mediated protection against acute liver injury (Supplementary Figure 1E). Accordingly, we quantified serum and hepatic levels of ceramide and its modified metabolite, lactosylceramide (Green et al., 2021). Our results demonstrated that APAP challenge (Model group) significantly elevated both ceramide and lactosylceramide levels in serum. Pretreatment with 3 g/kg or 1 g/kg PO dose-dependently suppressed this increase in ceramide and lactosylceramide (Figure 5A; Supplementary Figure 4A). Interestingly, while antibiotic-induced gut microbiota depletion (PGF + Model) did not significantly alter serum ceramide levels compared to the Model group, hepatic ceramide content was markedly reduced in PGF mice receiving PO pretreatment (PGF+3 g/kg PO) relative to both the Model and PGF + Model groups (Figures 5A,B).

Given that 16 S rDNA sequencing revealed PO-mediated modulation of lipid metabolism-associated gut microbiota, we hypothesized that PO regulates ceramide levels primarily through intestinal or microbial pathways (Zhou et al., 2025). To validate this finding, we measured ceramide levels in colonic tissues. Unlike serum and liver, colonic ceramide content remained unchanged in the Model group compared to controls. However, both 3 g/kg PO and PGF+3 g/kg PO groups exhibited significantly lower ceramide levels than their respective Model and PGF + Model counterparts (Figure 5C) (Ma et al., 2024). Collectively, these findings demonstrate that PO maybe modulates ceramide metabolism in a gut microbiota-dependent manner, influencing colonic ceramide levels, which in turn regulate systemic (serum) and hepatic ceramide dynamics. However, the current data are based mainly on total ceramide measurement, and the term “associated with changes in ceramide levels” is used rather than causal language.

Emerging evidence suggests that ceramide is closely associated with inflammation, oxidative stress, and the induction of ferroptosis (Chen et al., 2022; Zheng et al., 2025). Based on these findings, we hypothesized that elevated hepatic ceramide levels contribute to ferroptosis in the liver. Furthermore, ceramide has been shown to suppress NRF2 activation and expression, thereby influencing ferroptosis. Notably, NRF2 is a well-established therapeutic target for liver injury (Xu et al., 2024; Xu et al., 2022). To validate this hypothesis, we first measured levels of ferrous iron (Fe^2+^), the lipid peroxidation marker 4-hydroxynonenal (4-HNE), and ferritin (FE) in liver homogenates from different experimental groups. Our results revealed that the Model group exhibited a significant increase in Fe^2+^, 4-HNE, and FE levels. However, pretreatment with 3 g/kg and 1 g/kg PO effectively attenuated these elevations in a dose-dependent manner, indicating that APAP induces hepatic ferroptosis, while PO mitigates this process (Figures 5D–F,H) (Li et al., 2022; Liu et al., 2023). Prussian blue staining was also performed on paraffin sections of liver tissue, which further confirmed the hypothesis that severe iron ion deposition had occurred in the Model group. In contrast, this phenomenon of iron ion deposition was rescued to varying degrees in the treatment groups (Figure 5E).

To further elucidate the mechanism by which PO regulates ferroptosis, we examined the expression of several ferroptosis-related proteins, including FTH, TFRC, TFR2, NRF2, ACSL4, FPN1, KEAP1, NQO1, HO-1, P62, and GPX4. Our data demonstrated that the expression of these proteins was significantly altered in the Model group, and 3 g/kg PO treatment partially or completely reversed these changes (Figures 5G,I–O,T-V). Interestingly, the expression of SLC7A11, a key regulator of ferroptosis, remained unchanged in both the Model group and PO-treated groups (Figure 5S). Besides, we assessed the expression of key proteins involved in endoplasmic reticulum stress, NF-κB signaling (pP65, P65), energy metabolism (AMPK), and autophagy (P62, LC3B) (Figures 5P–R; Supplementary Figures 3C-F). These proteins also exhibited significant dysregulation in the Model group, which was effectively ameliorated by 3 g/kg PO treatment.

Collectively, these findings suggest that PO acts as a multi-pathway regulator, modulating diverse cellular signaling pathways, particularly those associated with ferroptosis.

### 3.7 PO attenuates APAP-Induced AML12 cell injury, ferroptosis and modulate ceramide metabolism

**FIGURE 6:**
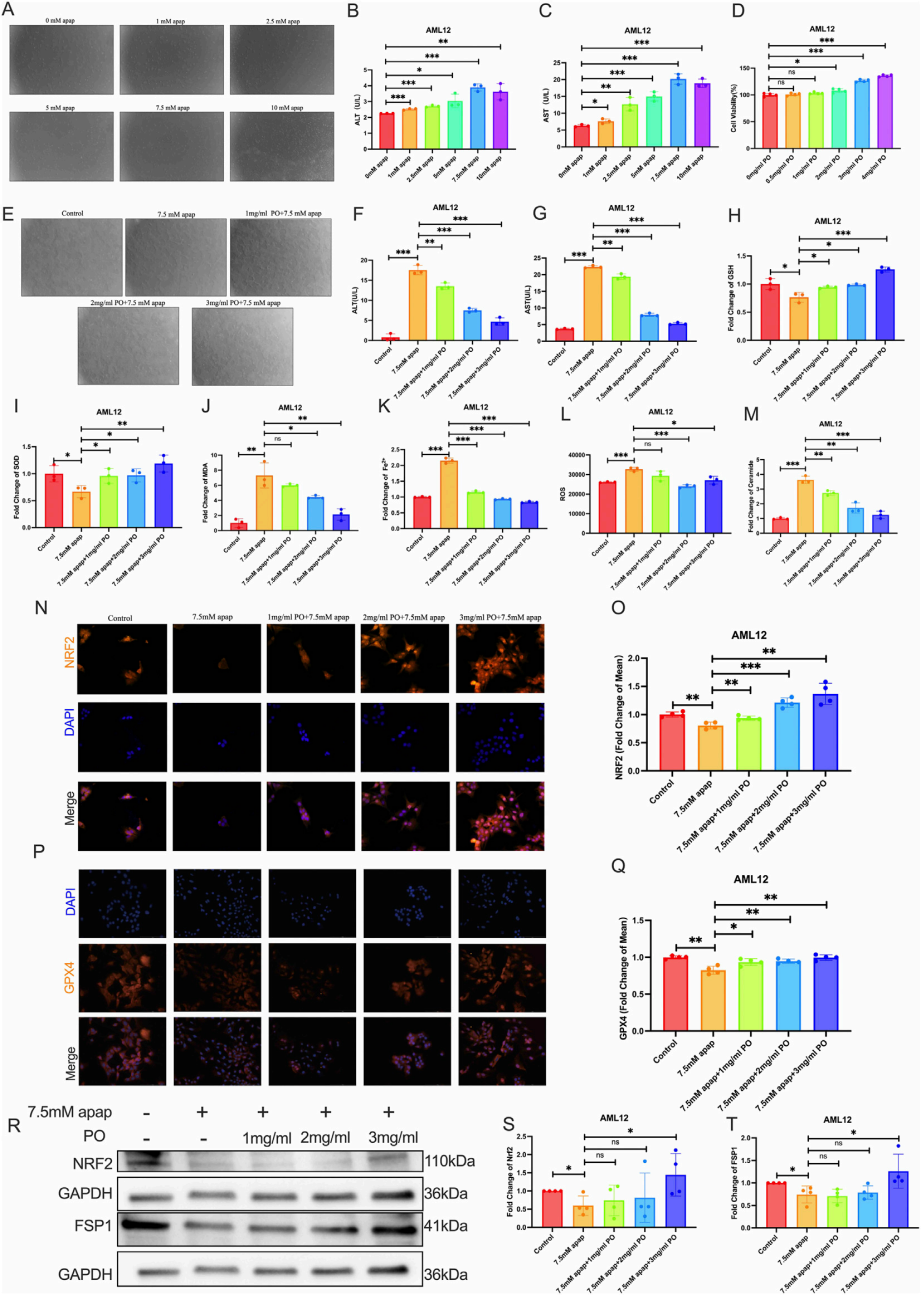
PO can alleviate APAP induced AML12 cell damage and ferroptosis. **(A)** Microscopic images of AML12 cells treated with different concentrations of APAP at 10 fold magnification. **(B,C)** Changes in AST and ALT levels in cell supernatant after 16 h of treatment with different concentrations of APAP. **(D)** CCK8 was used to detect the effect of different concentrations of PO on the viability of AML12 cells. **(E)** 10x AML12 cell microscopy images after different treatments. **(E–G)** Changes in AST and ALT levels in cell supernatant after 16 h of different treatments. **(H–J)** Changes in cellular oxidative stress indicators GSH, SOD, and MDA content after 16 h of different treatments. **(K)** Changes in cellular ferrous ion content after 16 h of different treatments. **(L)** Changes in cellular ROS after 16 h of different treatments. **(M)** Changes in cellular ceramide content after 16 h of different treatments. AML12 utilizes APAP for different treatments for 16 h, followed by NRF2 IF representative plots and statistical analysis of mean values. **(N,O)** AML12 cells treated with APAP for 16 h, followed by NRF2 immunofluorescence representative images and statistical analysis of mean values. **(P,Q)** AML12 utilizes different treatments with APAP for 16 h to generate representative graphs of GPX4 IF and perform statistical analysis on Mean values. **(R-T)** Representative graphs and relative quantitative analysis of NRF2 and FSP1 expression changes in cells after 16 h of different treatments using Western blot. Each dot represents a biological replicate. Statistical significance: *P < 0.05, **P < 0.01, ***P < 0.001 (compared between groups); “ns” indicates no significant difference.

To evaluate the protective effects of PO *in vitro*, we utilized AML12 cells as an experimental model (Qiu et al., 2023). Treatment with increasing concentrations of APAP for 16 h resulted in dose-dependent cell growth inhibition and death, accompanied by elevated levels of AST and ALT in the cell supernatant. The most pronounced cellular damage was observed at 7.5 mM APAP, which was consequently selected as the optimal concentration for subsequent experiments (Figures 6A–C).

**FIGURE 7:**
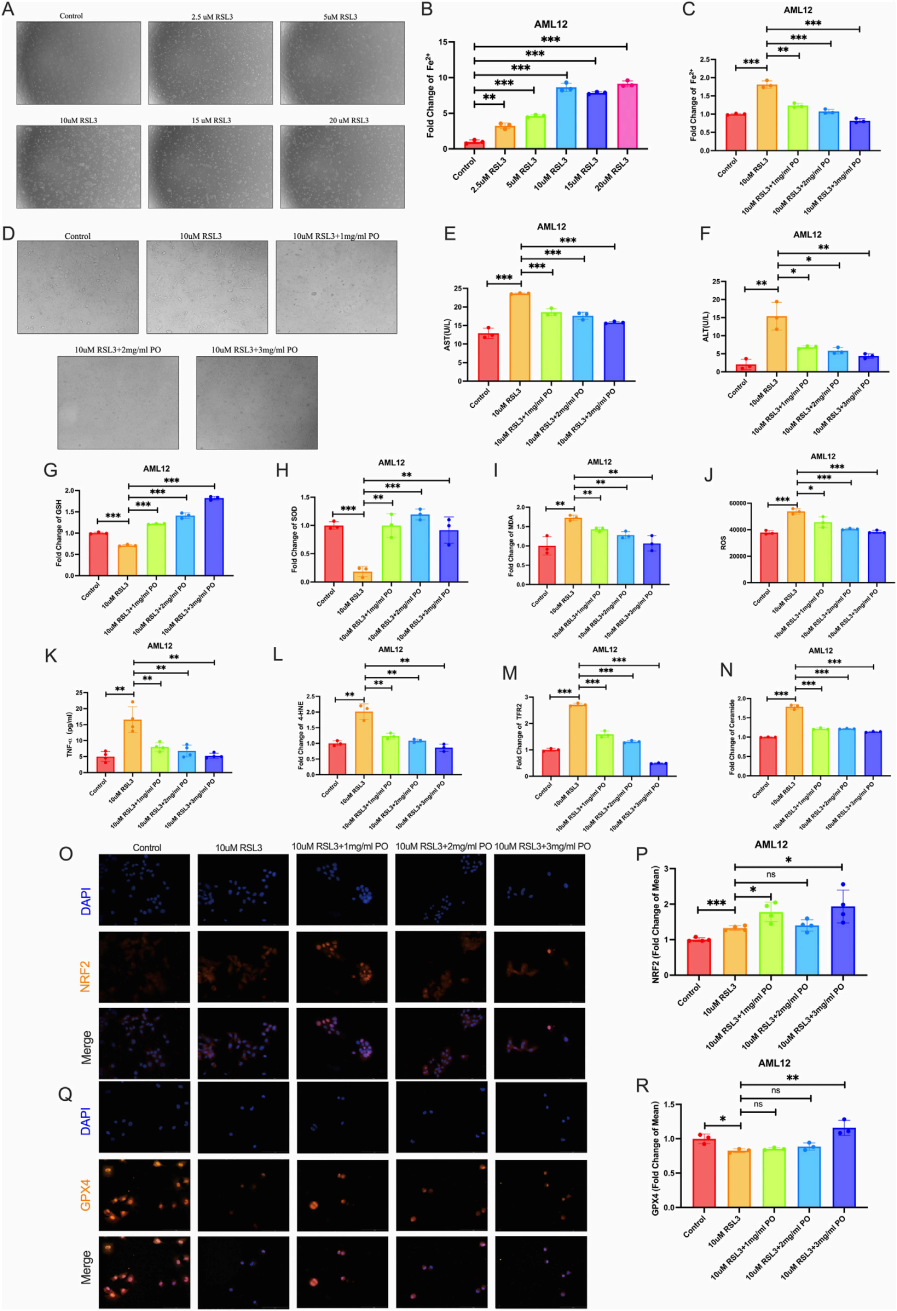
PO alleviates RSL3-induced AML12 cell injury and ferroptosis. **(A)** Microscopic images of AML12 cells treated with different concentrations of RSL3. **(B,C)** Changes in Fe^2+^ content in cells after 16 h of different treatments. **(D)** Microscopic images of AML12 cells under different treatments. **(E,F)** Changes in AST and ALT levels in cell supernatant after 16 h of different treatments. **(G–I)** Oxidative stress markers GSH, SOD, and MDA content in cells after 16 h of different treatments. **(J)** ROS levels in cells after 16 h of different treatments. **(K)** TNF-α content in cells after 16 h of different treatments. **(L)** Changes in 4-HNE content under different treatments. **(M)** TFR2 levels in cells after 16 h of different treatments. **(N)** Ceramide content in cells after 16 h of different treatments. **(O,P)** Representative immunofluorescence images and statistical analysis (mean values) of NRF2 in AML12 cells after 16 h of different treatments. **(Q,R)** Representative IF images and statistical analysis (mean values) of GPX4 in AML12 cells after 16 h of different treatments. Each dot represents a biological replicate. Statistical significance: *P < 0.05, **P < 0.01, ***P < 0.001 (compared between groups); “ns” indicates no significant difference.

To assess the impact of PO on cell viability, we performed CCK-8 assays and found that PO concentrations ranging from 2 to 4 mg/mL significantly enhanced cell survival (Figure 6D). Based on these findings, we selected three concentrations (2, 3, and 4 mg/mL) to investigate PO’s hepatoprotective effects against liver injury. Pretreatment with PO for 1 h prior to APAP exposure (7.5 mM, 16 h) markedly reduced APAP-induced cytotoxicity, as evidenced by decreased intercellular gaps and improved cell morphology. Furthermore, PO pretreatment dose-dependently attenuated the APAP-induced elevation of AST and ALT levels (Figures 6E–G). Analysis of oxidative stress markers revealed that APAP treatment significantly reduced GSH and SOD levels while increasing MDA. PO pretreatment effectively reversed these alterations in a dose-dependent manner (Figures 7H–J).

To examine whether ferroptosis contributes to APAP-induced cytotoxicity and whether PO alleviates this process, we measured intracellular Fe^2+^ and reactive ROS levels. Our results demonstrated that APAP treatment significantly increased Fe^2+^ and ROS accumulation, which was notably suppressed by PO pretreatment. However, only 2 and 3 mg/mL PO significantly inhibited ROS generation, but Fe^2+^ were dose-dependent inhibited by PO (Figures 6L,M). Besides, we quantified cellular ceramide levels and examined the expression and subcellular localization of NRF2 and GPX4 via immunofluorescence. Consistent with *in vivo* observations, APAP treatment elevated ceramide content, while PO dose-dependently mitigated this effect. Immunofluorescence analysis revealed attenuated NRF2 and GPX4 expression with reduced nuclear translocation of NRF2 following APAP exposure, all of which were partially restored by PO treatment (Figures 6M–Q).

Western blot analysis further confirmed that APAP significantly suppressed the expression of NRF2 and its downstream ferroptosis inhibitor FSP1, whereas 3 mg/mL PO substantially upregulated their expression (Figures 6R–T). Although pro-inflammatory cytokines IL-1beta and IL-6 were undetectable, APAP treatment markedly increased TNF-α secretion, which was dose-dependently inhibited by PO (Supplementary Figure 4D). Collectively, these findings demonstrate that PO exerts a direct hepatocyte-protective effect in AML12 cells, which may involve modulation of ceramide-associated NRF2/FSP1 or NRF2/GPX4 signaling. These *in vitro* observations are separate from the gut microbiota-dependent mechanisms observed *in vivo* (Ishii et al., 2022; Liang et al., 2023).

### 3.8 PO attenuates RSL3-Induced AML12 cell injury and ferroptosis by modulating ceramide metabolism

To further validate whether PO exerts its protective effects via ferroptosis inhibition, we employed RSL3, a potent ferroptosis inducer, in AML12 cells. Treatment with increasing concentrations of RSL3 for 16 h induced dose-dependent suppression of cell growth and viability, accompanied by a significant elevation in extracellular Fe^2+^ levels. Notably, Fe^2+^ accumulation reached near-maximal levels at 10 μM RSL3, which was thus selected as the optimal concentration for subsequent experiments (Figures 7A–B).

Consistent with our CCK-8 assay results, pretreatment with PO for 1 h prior to RSL3 exposure (10 μM, 16 h) markedly reduced cell death and increased cell density under microscopic observation. Moreover, PO dose-dependently attenuated the RSL3-induced upregulation of key ferroptosis markers, including Fe^2+^, 4-HNE, and TFR2. Similar trends were observed in supernatant levels of AST and ALT, which exhibited a positive correlation with Fe^2+^ accumulation (Figures 7C–F,L,M).

We further assessed oxidative stress markers and found that RSL3 treatment significantly reduced GSH levels and SOD activity, while increasing MDA levels. PO pretreatment partially reversed these alterations in a concentration-dependent manner (Figures 7G–I). In line with these findings, RSL3-induced overproduction of reactive ROS was robustly suppressed by PO (Figure 7J).

To explore the involvement of ceramide metabolism, we measured cellular ceramide levels and examined the expression of GPX4 and NRF2, a direct target of RSL3, via IF. RSL3 treatment elevated ceramide content, which PO mitigated in a dose-dependent manner (Figure 7N). IF analysis further confirmed that RSL3 suppressed GPX4 expression, whereas PO restored its levels (Figures 7Q–R). Intriguingly, unlike previous observations with APAP, RSL3 did not inhibit NRF2 expression. Instead, it upregulated NRF2, likely as a compensatory antioxidant response (Figures 7O–P). Notably, PO further enhanced NRF2 expression, particularly its nuclear translocation at 1 and 3 mg/mL, suggesting an augmented cellular antioxidant defense and potential downstream activation of GPX4.

### 3.9 Transcriptomics reveals how PO prevents APAP-Induced acute liver injury

To further elucidate the protective mechanism of PO against APAP-induced acute liver injury in mice, we performed RNA sequencing. Transcriptomic profiling revealed that 2,547 genes were differentially expressed in the Model (APAP-treated) group compared with the Control group (NC vs. M), with 1,506 upregulated and 1,041 downregulated. In contrast, 636 genes were significantly altered in the 3 g/kg PO-treated group compared to the Model group (M vs. H), comprising 343 upregulated and 293 downregulated genes. Volcano plots visualized the differentially expressed genes (DEGs) in both comparisons (NC vs. M and M vs. H), while a heatmap illustrated the top 50 DEGs in M vs. H that were also significantly altered in NC vs. M (Supplementary Figures 2A-D).

As a key inflammatory mediator, IL-6 was previously validated by ELISA, confirming the reliability of our transcriptomic dataset. Notably, DUOX2 emerged as the most significantly modulated gene in M vs. H among those already altered in NC vs. M (Supplementary Figure 2E). Given that DUOX1 and DUOX2 (members of the dual oxidase family) share structural and functional homology, and DUOX1/NF-κB signaling can be activated in a lipid raft-dependent manner, where ceramide is abundantly localized, we hypothesized that ceramide may similarly regulate hepatic DUOX2 (Bryndina et al., 2021; Wang et al., 2021; Wang et al., 2011).

IHC confirmed that DUOX2 expression was markedly elevated in the livers of APAP-treated mice, whereas 3 g/kg and 1 g/kg PO significantly suppressed its expression (Supplementary Figures 3A). *In vitro*, IF quantification in AML12 cells demonstrated that both 7.5 mM APAP and 10 µM RSL3 robustly increased DUOX2 levels, which were attenuated by PO in a dose-dependent manner (Supplementary Figures 3B-E). These findings not only validate the transcriptomic data but also suggest potential novel associations between ceramide, DUOX2, and ferroptosis.

Interestingly, despite the observed ferroptotic events (e.g., lipid peroxidation, GPX4 suppression), transcriptomics revealed no significant alterations in ferroptosis-related genes except for Acsl1 (a long-chain acyl-CoA synthetase) and Gstt3 (an NRF2-regulated detoxification enzyme). This suggests that ferroptosis in this context may be primarily regulated at the post-transcriptional or protein level (Jiang et al., 2021).

Transcriptomic sequencing and GO analysis demonstrated that in the Model (M) group, Biological Processes (BP) such as small molecule metabolic process, oxoacid metabolic process, and organic acid metabolic process were significantly altered. Molecular Functions (MF), including catalytic activity and oxidoreductase activity, as well as Cellular Components (CC), such as cytoplasm and extracellular region, were prominently affected. In contrast, comparisons between the high-dose PO treatment group (H, 3 g/kg PO) and the M group revealed perturbations in BP terms like nuclear division and organelle fission, MF terms related to extracellular functions, and CC terms including monooxygenase activity and oxidoreductase activity (acting on paired donors with incorporation or reduction of molecular oxygen) (Supplementary Figures 2F-H).

KEGG pathway analysis further identified significant alterations in the M group, particularly in Human Diseases (e.g., Chemical carcinogenesis - DNA adducts), Metabolism (e.g., Steroid hormone biosynthesis, Metabolism of xenobiotics by cytochrome P450), Organismal Systems (e.g., Bile secretion, PPAR signaling pathway), and Environmental Information Processing (e.g., Cytokine-cytokine receptor interaction, Viral protein interaction with cytokine and cytokine receptor). During the H vs. M comparison, key pathways impacted included Cellular Processes (e.g., Cell cycle, Oocyte meiosis, p53 signaling pathway), Organismal Systems (e.g., PPAR signaling pathway), Environmental Information Processing (e.g., Cytokine-cytokine receptor interaction), and Metabolism (e.g., Fatty acid degradation) (Supplementary Figures 2G-I).

Collectively, these findings indicated that PO administration rescues APAP-induced hepatic dysfunction. Notably, the p53 signaling pathway has been widely implicated in regulating the initiation and progression of ferroptosis, while the PPAR signaling pathway is closely associated with inflammation and oxidative stress (Wang et al., 2023; Wei et al., 2024). Thus, transcriptomic evidence suggests that PO likely exerts its hepatoprotective effects, including anti-inflammatory, antioxidant, and ferroptosis-inhibitory actions, primarily through modulation of the p53 and PPAR signaling pathways. Alternatively, by regulating the gut microbiota and/or ceramides to modulate these pathways, liver injury can be mitigated.

## 4 Discussion

Although N-acetylcysteine (NAC) remains the first-line clinical treatment for DILI due to its cost-effectiveness and accessibility (Akakpo et al., 2022), its therapeutic efficacy is limited by several factors: requirement for early high-dose administration, adverse effects (including bleeding, nausea, and gastrointestinal disturbances) (Coskun et al., 2021; Mao, 2023), and poor pharmacokinetic properties (low bioavailability and rapid clearance) (Coskun et al., 2021). In the present experimental design, both NAC and PO were administered as pretreatment before APAP challenge, which differs from the clinical use of NAC as an antidote after APAP overdose. Within this specific pretreatment setting, PO showed comparable or greater attenuation of liver injury markers at the tested concentrations, without observable toxicity. However, these findings should not be interpreted as clinical superiority of PO over NAC.

Ferroptosis, characterized by iron-dependent lipid peroxidation, involves three major regulatory pathways: iron metabolism, redox homeostasis, and lipid metabolism (Liang et al., 2022; Rochette et al., 2022; Yan et al., 2021). Contrary to previous reports, our study did not observe significant changes in SLC7A11 expression. Subsequent investigations suggested GPX4 modulation might occur through the NRF2/KEAP1 axis, though paradoxical findings were noted in downstream proteins (NQO1 and HO-1). This phenomenon may reflect compensatory upregulation of these proteins during severe ferroptosis in model groups, while treatment groups showed normalization approaching control levels. Furthermore, PO influenced iron metabolism proteins (FTH, TFRC, TFR2) and the lipid synthesis regulator ACSL4 (Chen et al., 2024; Guan et al., 2024), with additional experiments using the ferroptosis inducer RSL3 in AML12 cells, further supporting PO’s effects on ferroptosis-associated markers (Liu et al., 2024).

The NRF2 pathway reportedly plays a pivotal role in the regulation of oxidative stress, inflammation, and ferroptosis. Under basal conditions, cytoplasmic KEAP1 promotes NRF2 ubiquitination and proteasomal degradation (Adinolfi et al., 2023), while oxidative stress triggers NRF2 nuclear translocation. Our findings align with the existing literature, indicating that various hepatoprotective agents act through NRF2-mediated upregulation of NQO1 and HO-1 (Cai et al., 2022; Yang et al., 2023). Interestingly, although hepatic NRF2 protein levels remained unchanged, *in vitro* experiments revealed decreased nuclear translocation of NRF2, coinciding with increased ceramide levels, suggesting that ceramide may modulate NRF2 activity rather than expression (Kuo and Hla, 2024).

Sphingolipids, which act as intercellular second messengers, comprise a variety of species, including S1P and C1P (ceramide-1-phosphate) (Lin et al., 2025). Ceramides are the central metabolites in the sphingolipid signaling pathway. In this study, we only assessed the regulatory effect of PO on ceramides using 16 S rDNA sequencing and did not further investigate the changes in upstream and downstream sphingolipid metabolites. Moreover, ceramides exhibit different acyl chain lengths. The current consensus holds that C16:0- and C24:1-ceramides are the predominant species, and ceramides of varying chain lengths are thought to exert distinct physiological roles *in vivo* and *in vitro* (Gonzalez-Ramirez et al., 2024; Jingyi Bi, 2023).

A limitation of this study is that *in vivo* exposure information for PO-derived constituents remains unavailable. The currently identified components are based on extract profiling (UPLC-Q-TOF-MS), and it remains unclear which constituents or metabolites are absorbed, transformed by the gut microbiota, or delivered to liver tissue after oral administration. Therefore, the connection between the extract composition and the observed *in vivo* pharmacological effects should be interpreted with caution. Future pharmacokinetic and tissue distribution studies are warranted.

Polysaccharides from Polygonatum are one of the primary active components in Polygonatum extracts (Gong et al., 2024). Numerous studies have demonstrated that polysaccharides can modulate the gut microbiota and influence intestinal barrier function; therefore, polysaccharides may be one of the key components responsible for PO’s ability to regulate the gut microbiota (Chen et al., 2021; Ning et al., 2024).

Among the identified PO components, citric acid and ferulic acid were detected as major constituents. Citric acid, as a key intermediate in the tricarboxylic acid cycle, may contribute to energy metabolism regulation, while ferulic acid possesses well-documented antioxidant and anti-inflammatory properties. These constituents may partially underlie the observed hepatoprotective effects of PO, although the contributions of individual components require further investigation.

Transcriptomic analysis identified DUOX2 as a potential mediator of APAP-induced liver injury, with its activation possibly ceramide-dependent through lipid raft mechanisms, consistent with reported DUOX1 functions in the bronchial epithelium (Wang et al., 2011). PO treatment effectively suppressed DUOX2 overexpression, contributing to hepatoprotection.

Finally, we investigated the hepatoprotective effect of PO in female mice and found that pre-administration of 3 g/kg PO for 7 days significantly reduced the activities of AST, ALT, and LDH, indicating that PO still exhibits good hepatoprotective effects even in female mice (Supplementary Figures 4I-K). Overall, the above data substantiate that PO is a mixture with multiple targets and effects. PO can regulate the intestinal microbiota and is associated with changes in ceramide metabolism and ferroptosis-related markers. However, the causal relationships among these processes require further investigation.

As an extract derived from a medicinal and edible plant listed in China’s food-medicine homology catalog, PO has a long history of safe dietary use. This favorable safety profile, combined with its multi-target hepatoprotective effects demonstrated in this study, supports the potential of PO as a safe preventive supplement for liver health, although clinical studies are needed to confirm these findings in humans.

## Data Availability

The datasets presented in this study can be found in online repositories. The names of the repository/repositories and accession number(s) can be found below: The 16S rRNA amplicon sequencing data have been deposited in the NCBI Gene Expression Omnibus (GEO) under accession number GSE337417. The liver transcriptome sequencing data have been deposited in the NCBI Sequence Read Archive (SRA) under BioProject accession number PRJNA1494146.
