# Supplementary material for: Polygonatum odoratum extract ameliorates acetaminophen-induced acute liver injury by modulating the gut-liver axis and remodeling microbial and ceramide metabolism to suppress hepatic ferroptosis: insights from multi-omics
Source: Front Pharmacol. 2026 Jul 29;17:1907173. doi: 10.3389/fphar.2026.1907173 (PMC13461532; doi:10.3389/fphar.2026.1907173)
Supplement: Supplementary file 1 [file Supplementaryfile1.docx]

**Supplementary Figure_1 The active targets and enrichment pathways of PO in preventing APAP induced acute liver injury.** (A) Venn diagram of acute liver injury (ALI) related targets and PO targets. (B) Component disease target relationship network. (C) STRING mutual graph. (D) Drug compound target disease network diagram. (E) KEGG pathway analysis of target genes. (F) GO circle diagram of target gene. (G) GO enrichment analysis of target genes. (H) Pathway target KEGG enrichment analysis chart. The bubble size represents the number of genes in the pathway, and the color represents the p-value value.

**Supplementary Figure_2 Transcriptome sequencing further reveals the mechanism of PO action.** (A) Petal plot comparing different treatment groups. (B) Bar graph showing the number of upregulated and downregulated genes between comparison groups. (C) Volcano plot for NC (Control) vs. M (Model), highlighting the top 10 most significantly upregulated and downregulated genes. (D) Volcano plot for M (Model) vs. H (3g/kg PO), highlighting the top 10 most significantly upregulated and downregulated genes. (E) Heatmap of gene expression changes between groups, displaying the top 50 most significantly altered genes (25 upregulated, 25 downregulated) based on differential expression between model and control groups that were rescued by high-dose PO. (F-G) GO and KEGG enrichment analysis of differentially expressed genes (DEGs) in NC vs. M. (H-I) GO and KEGG enrichment analysis of DEGs in M vs. H (3g/kg PO). NC (Control): Normal control group;M (Model): Disease model group;H (3g/kg PO): High-dose PO treatment group (3g/kg).

**Supplementary Figure_3 DUOX2 is a key target of PO in preventing APAP-induced acute liver injury.** (A) Representative IHC (Immunohistochemistry) images of DUOX2 in liver paraffin sections across experimental groups. (B-C) IF representative images and quantitative mean fluorescence intensity analysis of DUOX2 in AML12 cells treated with APAP for 16 hours. (D-E) IF representative images and mean intensity analysis of DUOX2 in AML12 cells treated with RSL3 for 16 hours. Each dot represents a biological replicate. Statistical significance: *P < 0.05, **P < 0.01, ***P < 0.001 (compared between groups); "ns" indicates no significant difference.

**Supplementary Figure_4** (A) Serum lactosylceramide (LacCer) levels in mice across experimental groups. (B) Colon tissue homogenate Fe²⁺ content changes. (C) Western Blot representative images of PERK and AMPK expression in liver tissues. (D) TNF-α levels in AML12 cells after 16-hour APAP treatment. Quantitative analysis of liver PERK and AMPK expression. Serum biomarkers in female mice pretreated with 1/2/3 g/kg PO (L/M/H) for 7 days: AST,ALT,LDH2. Each dot represents a biological replicate. Statistical significance: *P < 0.05, **P < 0.01, ***P < 0.001 (compared between groups); "ns" indicates no significant difference.

**Supplementary Table 1:** The eight active ingredients identified through the screening process by TCMSP.

| [Mol ID](https://www.tcmsp-e.com/tcmspsearch.php?qr=Polygonati%20Odorati%20Rhizoma&qsr=herb_en_name&token=5263aea36f1f1ea1e6942d2e0b50949d) | [Molecule Name](https://www.tcmsp-e.com/tcmspsearch.php?qr=Polygonati%20Odorati%20Rhizoma&qsr=herb_en_name&token=5263aea36f1f1ea1e6942d2e0b50949d) | [OB (%)](https://www.tcmsp-e.com/tcmspsearch.php?qr=Polygonati%20Odorati%20Rhizoma&qsr=herb_en_name&token=5263aea36f1f1ea1e6942d2e0b50949d) | [DL](https://www.tcmsp-e.com/tcmspsearch.php?qr=Polygonati%20Odorati%20Rhizoma&qsr=herb_en_name&token=5263aea36f1f1ea1e6942d2e0b50949d) |
| --- | --- | --- | --- |
| MOL010387 | [3-o-beta-d-glucopyranosyl-(1-2)-[beta-d-xylopyranosyl-(1-3)]-beta-d-glucopyranosyl-(1-4)-galactopyranosyl-25(S)-spirost-5(6)-en-3beta,14alpho-diol_qt](https://www.tcmsp-e.com/molecule.php?qn=10387) | 104.58 | 0.79 |
| MOL010395 | [4',5,7-trihydroxy-6-methyl-8-methoxy-homoisoflavanone](https://www.tcmsp-e.com/molecule.php?qn=10395) | 89.7 | 0.33 |
| MOL010396 | [4',5,7-trihydroxy-6,8-dimethyl-homoisoflavanone](https://www.tcmsp-e.com/molecule.php?qn=10396) | 59.76 | 0.3 |
| MOL010408 | [polygosides E_qt](https://www.tcmsp-e.com/molecule.php?qn=10408) | 38.73 | 0.78 |
| MOL010411 | [4',5,7-trihydroxy-6-methyl-homoisoflavanone](https://www.tcmsp-e.com/molecule.php?qn=10411) | 82.94 | 0.27 |
| MOL010412 | [4'-methoxy-5,7-dihydroxy-6,8-dimethyl-homoisflavanone](https://www.tcmsp-e.com/molecule.php?qn=10412) | 57.14 | 0.34 |
| MOL000332 | [n-coumaroyltyramine](https://www.tcmsp-e.com/molecule.php?qn=332) | 85.63 | 0.2 |
| MOL000483 | [(Z)-3-(4-hydroxy-3-methoxy-phenyl)-N-[2-(4-hydroxyphenyl)ethyl]acrylamide](https://www.tcmsp-e.com/molecule.php?qn=483) | 118.35 | 0.26 |
